# A comprehensive evaluation of assembly scaffolding tools

**DOI:** 10.1186/gb-2014-15-3-r42

**Published:** 2014-03-03

**Authors:** Martin Hunt, Chris Newbold, Matthew Berriman, Thomas D Otto

**Affiliations:** 1 Parasite Genomics, Wellcome Trust Sanger Institute, Wellcome Trust Genome Campus, Cambridge CB10 1SA, UK; 2 Weatherall Institute of Molecular Medicine, University of Oxford, John Radcliffe Hospital, Oxford OX3 9DS, UK

## Abstract

**Background:**

Genome assembly is typically a two-stage process: contig assembly followed by the use of paired sequencing reads to join contigs into scaffolds. Scaffolds are usually the focus of reported assembly statistics; longer scaffolds greatly facilitate the use of genome sequences in downstream analyses, and it is appealing to present larger numbers as metrics of assembly performance. However, scaffolds are highly prone to errors, especially when generated using short reads, which can directly result in inflated assembly statistics.

**Results:**

Here we provide the first independent evaluation of scaffolding tools for second-generation sequencing data. We find large variations in the quality of results depending on the tool and dataset used. Even extremely simple test cases of perfect input, constructed to elucidate the behaviour of each algorithm, produced some surprising results. We further dissect the performance of the scaffolders using real and simulated sequencing data derived from the genomes of *Staphylococcus aureus*, *Rhodobacter sphaeroides*, *Plasmodium falciparum* and *Homo sapiens*. The results from simulated data are of high quality, with several of the tools producing perfect output. However, at least 10% of joins remains unidentified when using real data.

**Conclusions:**

The scaffolders vary in their usability, speed and number of correct and missed joins made between contigs. Results from real data highlight opportunities for further improvements of the tools. Overall, SGA, SOPRA and SSPACE generally outperform the other tools on our datasets. However, the quality of the results is highly dependent on the read mapper and genome complexity.

## Background

Obtaining a genome sequence is a vital component for detailed molecular analysis of an organism and for several thousands of species, genome projects are now underway or complete [1]. Through the process of *de novo* assembly, a genome is pieced together computationally, from overlapping randomly sequenced reads. Depending on several factors, including the depth of sequence coverage, sequencing methodology and complexity of the genome, the sequence can be assembled into a variable, but large number of contigs. The more fragmented the assembly is, the harder the downstream analysis becomes. Analysing gene order and synteny, carrying out comparative or functional genomics or investigating patterns of recombination all rely heavily on obtaining an assembly with good continuity.

Contig sizes are determined by the under-representation of sequences, due to coverage variation or sequencing bias, read lengths, sequencing technology and the existence of repeats in the genome. However, assembly continuity can be vastly improved by linking contigs together into scaffolds. This study compares the accuracy of methods that use paired sequences obtained from each end of DNA templates to bridge over regions of the genome that are difficult to sequence or assemble. Depending on the design of sequencing libraries, paired reads can bridge over gaps in sequence ranging from hundreds to tens of thousands of base pairs, to order and orientate contigs as well as to estimate the length of gaps. However, a combinatorial problem arises from repeat sequences. Repeats are often collapsed or mis-assembled during the assembly process, so that a scaffolder must resolve the numerous links arising from each repetitive contig. Unfortunately, scaffolding does not scale linearly and is computationally intractable [2].

The scaffolding problem can be formalised using graph theory, with contigs corresponding to nodes of the graph, and linking read pairs corresponding to edges. The available scaffolding tools take different approaches to produce approximate solutions. In addition to ordering and orientation, the expected distance between linking reads, known from the library construction, can be used to estimate the distance between contigs so that sequence gaps in the output scaffolds will be approximately of correct length. When used, this distance information is usually encoded into the scaffolding graph by assigning lengths to the edges. The ideal output from a scaffolder is one scaffold per chromosome of the genome, with gaps of the correct lengths separating each contig.

The increased availability of next generation sequencing (NGS) technology has driven intensive development of algorithms that assemble efficiently and, with varying success, scaffold millions of short sequencing reads into genome sequences. Recent studies that benchmarked *de novo* assemblers and analysed assembly quality, GAGE [3], Assemblathon [4] and Assemblathon2 [5], all reported large variations between assemblers, datasets and assembler parameters. Although most assemblers include a built in scaffolding algorithm, it is standard practice to perform an independent run of scaffolding immediately after *de novo* assembly to further improve continuity. Bambus [6], the first stand-alone scaffolding tool, was published in 2004 and consequently was not developed with large NGS datasets in mind. In this study we evaluated the contemporary scaffolders Bambus2 [7], GRASS [8], MIP [9], Opera [10], SCARPA [11], SOPRA [12] and SSPACE [13] and the scaffolding modules from the assemblers ABySS [14], SGA [15] and SOAPdenovo2 [16] (see Table 1). We have made all our scripts freely available so that the results can be reproduced and any new scaffolding tools can easily be analysed using our methods.

**Table 1:** Scaffolding tools

**Scaffolder**	**Version**	**Publication date**^**a**^	**Citations**^**b**^	**Method**	**Install**	**Run**	**Read mapper**	**Comments**
ABySS	1.3.6	27/02/2009	764^c^	Graph	+	o	User’s choice	
Bambus2	3.1.0^d^	16/09/2011	37	Graph	-	-	User’s choice	Hard to install and run. Scripting work needed to prepare input for running scaffolding
GRASS	0.003	06/04/2012	1	Graph	-	-	BWA/NovoAlign	Hard to install and did not produce scaffolds
MIP	0.5	13/10/2011	16	Graph	o	-	User’s choice	A few dependencies required, significant work needed to prepare input files
Opera	1.2	19/09/2011	30	Graph	+	o	Bowtie/BWA	
SCARPA	0.22	29/12/2012	3	Graph	+	o	User’s choice	
SGA	0.9.43	07/12/2011	74^c^	Graph	-	o	User’s choice	Several dependencies to install
SOAPdenovo2	r223	27/12/2012	47^c^	Graph	o	-	SOAP2	Little documentation on how to run scaffolder module alone
SOPRA	1.4.6	24/06/2010	41	Graph	+	-	User’s choice	Scripting work needed to prepare input for running scaffolding
SSPACE	2 (basic)	07/12/2010	151	Greedy	+	+	Bowtie/BWA^e^	Extremely easy to install and run

^a^Date the article was first available online.

^b^Retrieved from Google scholar 11 December 2013.

^c^Citations will include general assembly, not just scaffolding.

^d^Bambus2 is part of the AMOS package. AMOS version 3.1.0 was used, but with latest goBambus2 script from the AMOS git repository.

^e^BWA only available with a paid version of the software.

The first stage of any scaffolding algorithm is to decide where each read should be placed, if at all, in the input contigs. All the algorithms we tested use an existing read mapping tool to place reads in the assembly. Significant effort has been invested in tools that can efficiently map NGS sequencing reads to a genome sequence and there are many mappers now available [17]. Some scaffolders perform this read mapping for the user, by bundling one or both of the popular mappers BWA [18] or Bowtie [19] with the software, or in the case of SOAPdenovo2, use SOAP2′s own mapper. Other scaffolders require the user to run the mapping and provide a SAM or BAM [20] file of reads mapped to contigs, so that the user is free to choose any read mapper that outputs a compatible format.

Once the positions of reads in contigs have been determined, scaffolding can be undertaken. Bambus uses a greedy algorithm, joining together contigs with the most links first and ignoring subsequent edges that conflict with an existing join. SSPACE takes a similar approach, but starts building the first scaffold from the longest contig and continues to make joins as long as the majority of read pairs support the join. The other scaffolders do not use greedy algorithms but instead construct a graph and use a variety of methods to manipulate the graph and simplify the problem by partitioning the graph and solving each subgraph, or by changing the constraints on the graph.

Bambus2 first identifies repetitive contigs and removes them from the scaffolding graph, but retains contigs arising from variants, then orders and orients the contigs into scaffolds and finishes with a variant detection stage. SOPRA iteratively removes inconsistent linking information (edges of the graph) and contigs (nodes of the graph) that give rise to spurious links, using statistical optimisations to make the method practical. These steps are repeated until there are no more contigs to be removed and the graphs nodes and edges are all consistent. Opera solves the problem exactly, at the expense of dropping constraints on contig distances. Conversely, ABySS limits its search for inter-contig links using an upper limit on the allowed distance between any two contigs. SCARPA determines the orientation and then ordering of contigs in two separate stages, using restrictions that make the computation tractable and unlike the other scaffolders, includes a verification step that breaks contigs that appear to be mis-assembled. SGA uses a very conservative approach by essentially disallowing any conflicts in the graph, thereby avoiding heuristics at the expense of missing valid joins. MIP partitions the graph into subgraphs of restricted size and transforms each of these into a Mixed Integer Programming problem. Each subproblem is solved exactly because its size is limited and the constraint of contig orientation is not imposed. GRASS also uses Mixed Integer Programming but, like SOPRA, works iteratively to produce a final set of scaffolds.

## Results

**Figure 1:**
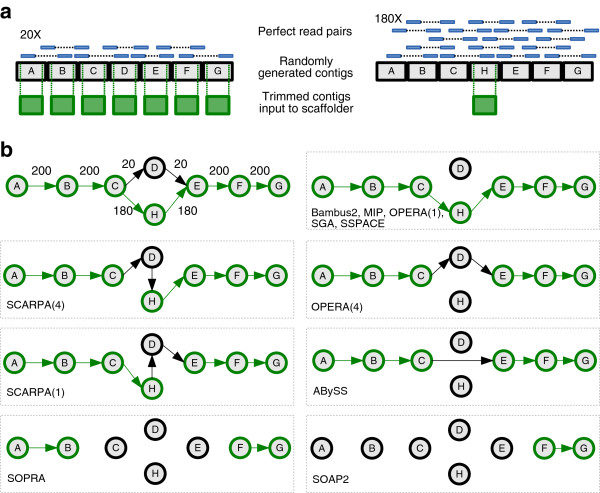
**Data generation and results of test case 11. (a)** Generation of contigs and read pairs for the test. **(b)** The test in graph form and the output of each scaffolder. Each node represents a 5 kb contig and each edge represents read pair evidence and is labelled with the read depth. Green nodes and edges mark the correct solution. Incorrect paths are coloured black. Numbers in brackets after each tool indicate the number of times that configuration was output by that tool. Tools with no number produced the same output on all runs.

We assessed the scaffolding tools using a variety of input data and found large variations in the results, depending on the scaffolder and mapping tool used, the genome being analysed and insert size of the library. In order to understand more about the behaviour of each scaffolder, we first constructed 11 simple test cases (Figure 1, Additional file 1: Figures S1-S3). We then went on to use larger datasets from four different genomes of varying size and GC content (Tables 2 and 3). These comprised Illumina reads from the *Staphylococcus aureus*, *Rhodobacter sphaeroides*, the human chromosome 14 GAGE datasets and the *Plasmodium falciparum* clone 3D7 reference genome [21]. Contig sets made by Velvet were chosen from the GAGE datasets because they were relatively more fragmented, maximising the possibility for the scaffolders to make joins. We included *P. falciparum* because it has an extremely low GC content of 19% and often poses quite a challenge to assembly algorithms despite its relatively small size of 23 MB. In addition to real data, four perfect datasets were generated from the *S. aureus* reference genome.

**Table 2:** Summary of datasets

**Dataset**	**Size (Mb)**	**%GC**	**Potential joins**	**Number of reads (millions)**	**Read length**	**Insert size**^**a**^	**Percent reads mapped** (**fwd/rev)**								**Maximum read coverage**
							**Bowtie -v 0**		**Bowtie -v 3**		**Bowtie2**		**BWA**		
*S. aureus* perfect	2.8	32	27	0.76	76	505	96	96	95	95	97	97	97	97	40
			27	0.76	76	2,795	96	96	95	95	97	97	97	97	40
			940	0.76	76	505	95	94	94	94	96	96	96	96	40
			940	0.76	76	2,995	95	94	94	94	97	96	96	96	40
*S. aureus* GAGE	2.9	32	167	3.5	37	3,385	35	35	56	54	55	54	53	52	49
*R. sphaeroides* GAGE	4.6	68	570	2.1	101	3,695	17	11	36	32	69	68	35	30	62
*P. falciparum* 3D7 de novo	23.3	19	9,302	52.5	76	645	70	67	73	70	79	77	76	74	267
			9,302	12.0	75	2,705	27	25	31	30	43	42	33	32	33
Human chromosome 14 GAGE^b^	88.2	40	19,935	22.7	101	2,865	46	19	68	29	90	55	69	30	38
			19,935	2.4	57-82	34,500	47	6	73	48	93	85	79	56	3

^a^Calculated from Bowtie2 mapping to contigs.

^b^Excluding the 19 Mb of Ns at the start of the sequence.

**Table 3:** Results summary of simulated data

**Scaffolder**	**Read mapper**	**10 kb contigs**^**a**^				**3 kb contigs**					
		**Short fragment reads**		**Long fragment reads**		**Short fragment reads**			**Long fragment reads**		
		**% Correct joins found**	**Score**	**% Correct joins found**	**Score**	**% Correct joins found**	**% Joins incorrect**	**Score**	**% Correct joins found**	**% Joins incorrect**	**Score**
ABySS	abyss-map	100.0	1.00	66.7	0.91	98.9	0.0	1.00	99.5	0.0	1.00
Bambus2	Bowtie 2	100.0	1.00	66.7	0.91	63.7	0.0	0.84	95.9	0.0	0.99
	BWA	48.2	0.82	48.2	0.87	59.6	0.0	0.82	96.2	0.0	0.99
MIP	Bowtie -v 0	100.0	1.00	96.3	0.99	98.9	0.0	1.00	98.4	0.0	1.00
	Bowtie -v 3	100.0	1.00	96.3	0.99	98.4	0.0	0.99	97.9	0.0	1.00
	Bowtie 2	100.0	1.00	29.6	0.82	96.3	0.5	0.74	98.7	0.5	0.96
	BWA	100.0	1.00	33.3	0.83	98.0	1.1	0.54	98.2	0.4	0.96
Opera	Bowtie	100.0	1.00	92.6	0.98	98.4	0.0	0.99	1.0	10.0	0.79
	BWA	100.0	1.00	92.6	0.98	99.8	0.2	0.93	1.2	80.0	0.37
SCARPA	Bowtie -v 0	100.0	1.00	96.3	0.99	98.9	0.0	1.00	95.0	0.0	0.99
	Bowtie -v 3	100.0	1.00	96.3	0.99	98.6	0.0	0.99	96.3	0.0	0.99
	Bowtie 2	85.2	0.95	96.3	0.99	96.8	0.0	0.99	76.3	0.7	0.92
	BWA	85.2	0.95	92.6	0.98	96.6	0.0	0.98	77.9	0.4	0.93
SGA	Bowtie 2	100.0	1.00	96.3	0.99	97.3	0.0	0.99	97.6	0.0	1.00
	BWA	100.0	1.00	92.6	0.98	99.0	0.0	1.00	96.2	0.0	0.99
SOAP2	SOAP2	96.3	0.99	96.3	0.99	98.6	0.0	0.99	99.5	0.0	1.00
SOPRA	Bowtie -v 0	100.0	1.00	96.3	0.99	98.3	0.0	0.99	98.2	0.0	1.00
	Bowtie -v 3	100.0	1.00	96.3	0.99	97.2	0.0	0.99	97.2	0.0	1.00
	Bowtie 2	74.1	0.91	100.0	1.00	91.5	0.5	0.82	85.6	0.5	0.93
	BWA	74.1	0.91	88.9	0.97	92.9	0.2	0.89	83.1	0.4	0.93
SSPACE	Bowtie -v 0	100.0	1.00	92.6	0.98	99.1	0.0	1.00	99.6	0.0	1.00
	Bowtie -v 3	100.0	1.00	92.6	0.98	98.7	0.0	0.99	99.3	0.0	1.00

^a^No incorrect joins were made by any of the tools.

GRASS did not join any contigs together in any of the test cases or using the *S. aureus* genome. It either crashed or wrote ‘scaffolds’ that simply consisted of the input contig sequences. Therefore it was not analysed any further.

### Read mapping

As described earlier, some of the scaffolders run the read mapping for the user using either BWA or Bowtie (except for SOAPdenovo2). Every scaffolder was run using its default settings. However Opera and SSPACE run Bowtie using slightly different settings with one more permissive than the other (see Materials and methods for details). To be consistent, for each scaffolder wherever possible both settings of Bowtie were used in addition to one run each with BWA and Bowtie2 [22], so that up to four runs of each scaffolder were carried out. Reads were mapped for ABySS using its own mapper (abyss-map) with the default settings.

The way in which the read mappers were run meant that BWA maps a read if there were up to two mismatches in its first 32 bases and if it could map to more than one position, one of those positions was chosen at random. Bowtie2 also places repetitive reads randomly but only reports reads mapped with a minimum alignment score that depends on the length of the read. Bowtie mapped any read that matched to only one position, and either had no mismatches (when using the option -v 0) or up to three mismatches (when using -v 3). Table 2 and Additional file 2: Table S1 show the difference in numbers of reads mapped for each dataset and read mapper. For example, on human chromosome 14 Bowtie -v 0 mapped just 6% of the reverse read of each pair, whereas Bowtie2 mapped 85% of those reads. However, because more relaxed mapping parameters produce more erroneously mapped reads, the number of mapped reads does not correlate with scaffolding accuracy.

All the scaffolders except SGA and SOAPdenovo2 need the forward and reverse paired reads to be independently mapped to contigs and only after mapping is the fact that each read has a mate used as linking information. SGA is an exception because it requires the user to map the reads as true paired reads. However, Bowtie does not report reads within a pair that map to different contigs and so yields no scaffolding information. For this reason, only Bowtie2 and BWA were used to map reads when running SGA. Although Bambus2 can take linking data from any source, it was non-trivial to convert the linking information from mapped reads into the required format. For this reason only Bowtie2 and BWA were used with Bambus2, since a BAM file could be converted using a script from the ABySS package.

### Evaluation metrics

**Figure 2:**
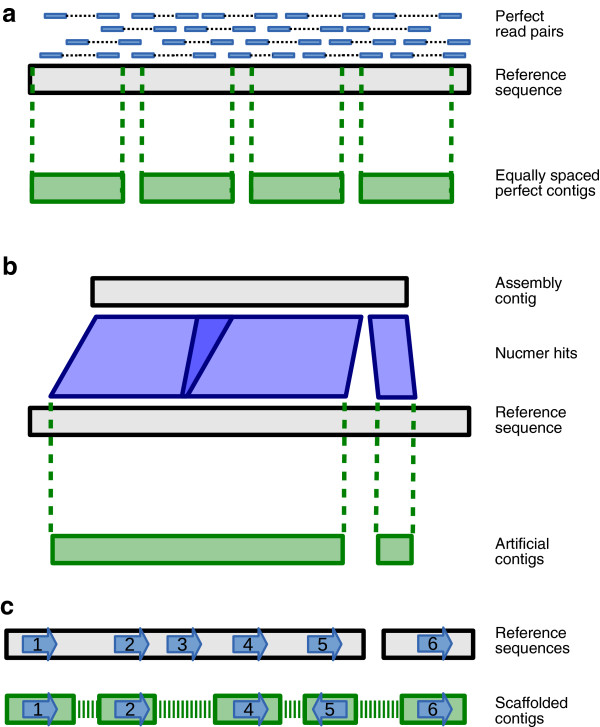
**Simulated contigs, artificial contigs and sequence tags. (a)** Generation of simulated contigs and reads from the *S. aureus* reference sequence. **(b)** Generation of artificial contigs from assembler output. **(c)** Tag types. Tags 1 and 2 are a correct join. Tags 2 and 4 demonstrate a skipped tag because the output scaffold jumps over tag 3. Tag 3 also does not appear in the output and is therefore a lost tag. Tags 4 and 5 are in the wrong orientation and tags 5 and 6 belong to different sequences in the reference.

To test the accuracy of each scaffolding algorithm in detail, we generated test contigs that contained no errors and could be tracked using unique sequence tags (see Materials and methods, Figure 2 and Additional file 1: Figure S5), but still resembled real contigs that would be produced by an assembler. Since the tags were unique within the dataset they allowed the order, orientation and distance of the contigs to be determined within the scaffolds output by each tool.

The quality of each scaffolder was assessed using the following five key metrics (see Figure 2 and Materials and methods for a complete explanation).

• Correct joins - a pair of contigs correctly joined with the tags in the expected orientation and separated by the correct distance.

• Incorrect joins - where contigs from different locations, in the wrong orientation or separated by the incorrect distance were joined together.

• Skipped tags - two contigs were correctly joined, but separated by a gap that should contain another contig.

• Lost tags - a tag that was completely absent from the output of the scaffolder.

• Running time - the total CPU time used, including any pre-processing stages and read mapping.

Within each dataset, a score between 0 and 1 was calculated for each metric by scaling the scores so that the best tool scored 1 and the worst scored zero. For example, taking correct joins, the tool that produced the most correct joins scored 1, the tool that made the fewest correct joins scored 0 and the scores of the other tools were scaled between those values. Within each dataset, a single summary score, between 0 and 1, was calculated for each tool by summing weighted scores and dividing by the total of the weights. For example, weighting all five metrics equally with a weight of 1 would mean summing the scores and then dividing by 5. Note that an overall score of 1 for a particular tool and dataset does not mean that it produced the ideal answer, but that it was not outperformed by another tool.

A dynamic Excel spreadsheet is provided in Additional file 2: Table S2, where summary scores automatically update depending on the choice of weights, allowing different weighting schemes to be explored. A distribution of summary scores for each tool on each dataset was obtained by weighting the metrics with a range of values intended to emphasise accuracy (see Materials and methods for details). In addition to considering combinations of weights, we singled out the weighting of 80, 160, 160, 40 and 1 for correct joins, incorrect joins, lost tags, skipped tags and running time, which represents our opinion of the relative importance of the metrics and heavily penalises errors.

### Small test cases

The 11 test cases consisted of simple input and used simulated perfect Illumina read pairs together with contigs of random sequence and neutral GC content. This revealed the different approaches that were taken by each scaffolder depending on the available choices (Figure 1, Additional file 1: Figures S1-S3). Each test was repeated five times, with an independently generated set of input data, to account for any randomness within the methods of the tools. We expected that all of the scaffolders would solve these tests in a sensible way by using the majority of read pair evidence whenever possible and would not make any obvious errors. Figure 1, which is test 11 in Additional file 1, exemplifies these tests, where most of the joins between contigs are unambiguous but a scaffolder must choose between two options for other joins.

The simplest part of test 11 was to join contigs A-B-C and E-F-G, where there was high supporting read coverage and no ambiguities (see also tests 1 and 2 in Additional file 1). All scaffolders except SOPRA and SOAPdenovo2 successfully made these joins. The remaining differences in behaviour demonstrated how the tools dealt with the choice between C-D-E or C-H-E, with the latter option having nine times more read pair evidence. First, we note that SCARPA generated errors by joining either C-D-H-E or C-H-D-E and this behaviour was consistent throughout all similar tests (Supplementary tests 6–10). Opera chose either C-D-E or C-H-E, demonstrating that it did not make use of the read coverage to resolve conflicts but instead chose seemingly at random.

In every test, Bambus2, MIP, SGA and SSPACE all elected to follow the path of most evidence, when present, but differed when there was equal support for two choices. In test 9, which only differs from test 11 by having equal read pair evidence for C-D-E and C-H-E (see Additional file 1: Figure S3), SSPACE stopped scaffolding at the ambiguity by not placing D or H in a scaffold. MIP chose the same path in all five iterations, suggesting that it uses some heuristic to make its choice. SGA appeared to choose its path randomly, outputting either C-D-E or C-H-E in the five iterations. ABySS skipped D and H, choosing C-E instead, suggesting that ABySS knew the scaffolding path, but decided to skip the nodes because of the heterozygosity.

SOPRA and SSPACE behaved predictably on all the test cases, with both tools consistently making no joins whenever there were two options with equal evidence. Bambus2 behaved similarly to SSPACE, with two exceptions (tests 2 and 6). However, SOPRA made fewer joins than SSPACE and the most likely explanation for this is that its algorithm involves removing nodes (contigs) from the graph as well as edges (read pair links). This means that a contig that could be joined to more than two other contigs (for example contig C in Figure 1a) would be removed from the graph and so not placed in a scaffold.

### Simulated datasets

Two sets of simulated contigs and reads were generated from the *S. aureus* reference genome that was used in the GAGE study (Figure 2a), excluding its two plasmid sequences. Perfect contigs of lengths 3 kb and 10 kb, separated by gaps of 50 bp and 300 bp, respectively, were made. Perfect Illumina read pairs were sampled from the reference sequence with uniform coverage and mean insert size of 500 bp and 3 kb. The combinations of perfect reads and contigs gave four datasets for testing the scaffolders.

Overall, the tools performed well on the simulated datasets, with many tools reconstructing the entire sequence correctly (Table 3, Additional file 2: Table S3, Additional file 1: Figures S6-S9). However, each dataset had at least one combination of tool, mapper and mapping parameters that performed badly. The simplest test was that which used 10 kb contigs with simulated libraries that contained 500 bp inserts. Every tool produced the correct answer with at least one read mapper, making every available join between contigs with no errors. The most notable results on this dataset were those of Bambus2 and SOPRA, both of which showed large variations depending on the read mapper. SOPRA produced a perfect result if Bowtie was used to map the reads, but missed 26% of the potential joins if Bowtie2 or BWA was used instead. As can be seen in Table 2, Bowtie2 and BWA mapped more reads than Bowtie demonstrating that more mapped reads does not necessarily lead to better scaffolds. Using the same contigs but simulated libraries with a 3 kb insert size resulted in slightly worse output, with most tools making 25 or 26 of the possible 27 joins. This time SOPRA with Bowtie2 was the only scaffolding run that produced a completely perfect result.

Using the 3 kb contigs there were 940 potential contig joins that could be made, and this posed more of a challenge to the scaffolders, particularly using the 3 kb insert reads (Table 3). Excluding Bambus2 and Opera, on average, 97% of the possible joins were correctly made by each tool when using the 500 bp insert data. When the 3 kb insert reads were used instead, this number dropped to 94%. In contrast, Bambus2 found 62% of joins using the short insert data, but this number increased to 96% on the long insert reads. Opera only identified 1% of the joins from the long insert reads.

The effect of read mapping tools and parameters is already visible in the simulated data. For example, the percent of perfect joins found by SCARPA on the 3 kb contigs with 3 kb insert reads ranged from 76% to 96%, depending on the read mapping parameters (Table 3). SOPRA’s values ranged from 91% to 98% when using the 500 bp insert reads. Moreover, for both tools the run that found the most correct joins was also the most accurate, making no incorrect joins.

### Genome scale data

The results of scaffolding realistic data are presented below, broken down by dataset. In each case, real sequencing read pairs were used to scaffold artificial contigs generated from real genome assemblies, constructed as follows (see Materials and methods and Figure 2b for complete details). Each real contig from a genome assembly was aligned to the reference genome and the sequence of each match in the reference was used as an artificial contig. Any artificial contigs with sequence overlap that were generated from the same assembly contig were merged. It was necessary to use artificial contigs in order to track their placement in the output from each scaffolder.

Summary data and plots of the *P. falciparum*, *S. aureus* and human datasets are given in Figure 3 and Table 4 (see Additional file 2: Table S3 and Additional file 1: Figures S10-S17 for complete data and plots).

**Figure 3:**
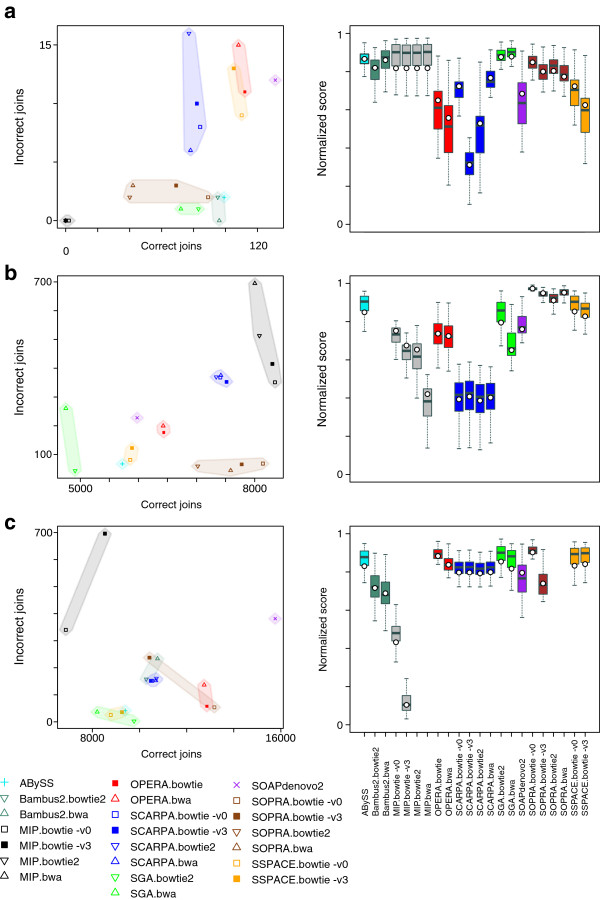
**Genome-scale data results. (a)***S. aureus* GAGE data, **(b)***P. falciparum* combined short and long data and **(c)** human chromosome 14 combined short and long insert data. Scatterplots show the relationship between correct and incorrect joins made by each scaffolder. Boxplots show the distribution of summary scores when iterating over different score combinations. The white circles in the boxplots denote the score from our chosen weighting system that focuses on penalising errors (with weights: correct join = 80, incorrect join = 160, lost tag = 160, skipped tag = 40, running time = 1).

**Table 4:** Results summary of genome scale data

**Scaffolder**	**Read mapper**	***S. aureus***			***P. falciparum***			**Human chromosome 14**		
		**% Correct joins found**	**% Joins incorrect**	**Score**	**% Correct joins found**	**% Joins incorrect**	**Score**	**% Correct joins found**	**% Joins incorrect**	**Score**
ABySS	abyss-map	59.3	2.0	0.87	61.5	1.2	0.85	47.1	0.5	0.83
Bambus2	Bowtie 2	56.9	2.1	0.82	NA^a^	NA^a^	NA^a^	51.6	1.5	0.72
	BWA	57.5	0.0	0.86	NA^a^	NA^a^	NA^a^	54.0	2.1	0.69
MIP	Bowtie -v 0	1.2	0.0	0.82	89.8	4.0	0.75	34.5	4.7	0.43
	Bowtie -v 3	0.0	NA	0.82	89.3	4.8	0.68	42.8	7.5	0.10
	Bowtie 2	0.0	NA	0.82	86.9	6.0	0.65	NA^a^	NA^a^	NA^a^
	BWA	0.0	NA	0.82	86.0	8.0	0.42	NA^a^	NA^a^	NA^a^
Opera	Bowtie	67.1	8.9	0.65	69.2	2.7	0.74	64.5	0.4	0.88
	BWA	64.7	12.2	0.56	69.1	3.0	0.72	63.9	1.1	0.84
SCARPA	Bowtie -v 0	50.3	8.7	0.72	79.6	4.7	0.39	52.8	1.4	0.80
	Bowtie -v 3	49.1	10.9	0.31	80.8	4.5	0.41	52.6	1.4	0.80
	Bowtie 2	46.1	17.2	0.53	78.9	4.8	0.39	53.7	1.5	0.79
	BWA	46.7	7.1	0.77	79.7	4.8	0.40	53.6	1.4	0.80
SGA	Bowtie 2	49.7	1.2	0.88	52.8	0.9	0.80	49.0	0.0	0.85
	BWA	43.1	1.4	0.88	51.1	5.2	0.65	41.1	0.4	0.82
SOAP2	SOAP2	78.4	8.4	0.68	64.3	3.7	0.76	79.0	2.4	0.80
SOPRA	Bowtie -v 0	53.3	2.2	0.85	87.5	0.8	0.97	66.1	0.4	0.90
	Bowtie -v 3	41.3	4.2	0.80	83.6	0.8	0.95	52.3	2.2	0.74
	Bowtie 2	24.0	4.8	0.80	75.4	0.8	0.91	NA^a^	NA^a^	NA^a^
	BWA	25.1	6.7	0.77	81.5	0.6	0.95	NA^a^	NA^a^	NA^a^
SSPACE	Bowtie -v 0	65.9	7.6	0.72	63.0	1.4	0.85	44.0	0.3	0.83
	Bowtie -v 3	62.9	11.0	0.62	63.3	2.0	0.83	46.4	0.4	0.84

^a^Data not available because scaffolder required more than 30 GB of memory or did not finish within 12 days.

#### S. aureus

The ‘shortjump’ reads, with a fragment size of approximately 3.4 kb, were used to scaffold artificial contigs generated from the Velvet [23] contigs of the GAGE *S. aureus* dataset. There were 167 potential joins from the input contigs and the proportion of those that were found correctly ranged from almost no joins up to 78%, with an average of 51%. This average value excludes MIP because on this dataset it failed to join almost any contigs, regardless of the mapper used. This flattered its apparent quality when considering different combinations of scoring weights (Figure 3a) because no joins meant that it made no errors. Excluding MIP, the most conservative tools were SGA and Bambus2 (Figure 3a). SGA found 43% to 50% of the joins depending on the mapper, with an error rate of 1%. Bambus2 had one run where it found 57% of the potential joins with no false positives. At the other extreme, SOAPdenovo2 correctly identified 78% of the joins at the cost of an 8% error rate (Figure 3a), which was the average across all tools (excluding MIP). Every scaffolder skipped over some possible joins, with Opera having the most skipped tags (20).

Overall, using our specific scoring that penalises errors (Box plot Figure 3a and Additional file 2: Table S3), SGA, ABySS and Bambus2 generated the best results. However, the boxplots also show that, depending on the scoring system, the results do change considerably.

The dependence on the mapping is immediately evident from Figure 3a. The error rates of SCARPA, SSPACE and Opera were affected by the mapping, but they showed relatively little variation in the number of correct joins. On the other hand, the number of correct joins made by SOPRA showed more variation than the error rate. On this dataset Bowtie with no mismatches (−v 0) performed better than with three mismatches (−v 3) on the tools where this could be changed (MIP, SCARPA, SOPRA and SSPACE).

#### R. sphaeroides

The complete results for this dataset are given in the Additional file 1: Figure S11 and (Additional file 2: Tables S2 and S3). However we remark here that similarly to *S. aureus*, SGA was the most conservative, SOAPdenovo2 was the most aggressive, with a better than average error rate, and there was a heavy dependence on the mapping parameters. This is the only dataset on which SCARPA performed particularly well when compared with the other scaffolders.

#### P. falciparum

Contigs were made for input to the scaffolders using *de novo* assembly contigs from one set of short fragment (645 bp) Illumina read pairs. The short fragment read pairs and a second set of Illumina reads with a fragment size of 2.7 kb were used for scaffolding. This meant that we could test the scaffolders in three different ways, by using each read set on its own and then a third run using both sets together.

SSPACE made best use of the short fragment reads alone, finding 62% of the potential 9,230 joins, but SOAPdenovo2 and SOPRA were comparable (Additional file 2: Tables S2 and S3, Additional file 1: Figure S12) and all three scaffolders had error rates of 1% to 3%. However, SOPRA made the best use of the large fragment reads (Additional file 2: Tables S2 and S3, Additional file 1: Figure S13) therefore it was the most accurate on two read sets combined (Figure 3b) striking the best balance between making joins and minimising errors. The most aggressive settings with this tool found 87% of the joins, with an error rate under 1%. This compares with an average of 75% of correct joins and a 3% error rate. SGA was again the most conservative on this dataset, finding 53% of the joins and only 44 errors (<1%) with its best run. However, the most accurate run using SOPRA made 45 errors and correctly identified 82% of the potential joins and had the highest overall score of 0.97. This result still comprised over 1,000 scaffolds from what should be 16 sequences. Both runs of Bambus2 failed on this dataset, one due to exceeding our limit of 12 days run time and the other when its memory usage reached the machine limit of 30 GB.

The proportion of skipped and lost tags becomes noticeable in this dataset for some of the tools, particularly when using both sets of reads. SCARPA lost 2% to 3% of the 9,318 tags and had similar numbers of skipped tags and MIP lost up to 1% of the tags. Opera had approximately 1,170 skipped tags, which means that 18% of the 6,430 joins it made jumped over at least one contig. This explains the high corrected N50 of Opera (Additional file 2: Table S3), as it ignores smaller contigs. Although some users might want scaffolds with a large N50, this metric does not necessarily reflect correctly orientated contigs in a scaffold.

This dataset demonstrates the effectiveness of using libraries of different fragment sizes, as is common practice in a genome project. The percent of potential joins correctly made using just the short fragment reads averaged at 53% across all runs, and increased to 59% using the long fragment reads. When both sets were used together the average becomes 75%, with MIP achieving 89% albeit with a high error rate of 8% to 10%.

On this dataset the mapping options appeared to be less significant than on others, with SSPACE, SCARPA and SOPRA showing little difference between their runs. This suggests that the different mappers behaved similarly and indeed the number of reads mapped showed little variation compared to the other real datasets (Table 2). As was the case for the *S. aureus* Velvet contigs, the number correct joins made by SOPRA was affected more than its error rate. The opposite was true of MIP, with mapping settings causing its error rate to vary but relatively small changes were seen in the number of correct joins. It is notable that BWA did not map the short insert reads accurately to the extremely low GC content genome of *P. falciparum* and introduced many errors or prevented scaffolding with Bambus2 and SGA. Bowtie2 is clearly the better choice on this genome for these tools.

#### H. sapiens

The Velvet contigs from human chromosome 14 from the GAGE data were scaffolded with the ‘short jump’ and ‘long jump’ libraries, of fragment length 3 kb and 35 kb, respectively. Similarly to the *P. falciparum* data, this meant that three scaffolding runs could be performed using each read set separately or combined. On average, 57% of the joins were found when using only the short jump library and 13% using the long jump library, showing that the long jump library was difficult to use successfully possibly because it was of low quality and relatively low read coverage (Table 2). Some runs of Bambus2, SOPRA, SSPACE, MIP and SGA produced worse results on the combined dataset than on just the short jump reads. The net effect of this was that an average of 53% of the joins were successfully found using the combined read set, which was less than that of the short jump reads.

As was the case for *P. falciparum*, SOPRA made best use of combining the short and long fragment reads, successfully joining 66% of the potential 19,935 joins with an error rate of 0.4%. However, it was not such a clear winner and was comparable to Opera, which had the same error rate as SOPRA and made 65% of the joins. Again, SGA was the most conservative, making 49% of the joins correctly with just three errors, but SSPACE produced scaffolds of a similar quality. SOAPdenovo2 was the most aggressive, identifying 79% of the potential joins with a 2% error rate. The range of behaviour, from conservative scaffolding with a small number of errors to aggressive scaffolding, can be seen in the scatterplot in Figure 3c. The boxplot of Figure 3c shows that our scoring (white circles) is generally more conservative and the tools are more error-prone on this dataset.

The use of the 35 kb fragment size data led to a large number of skipped tags. On average each tool made 10,757 joins using the combined set of reads, but skipped 2,836 tags. However, considering just the large fragment reads, there was an average of 2,828 joins and 2,212 skipped tags, which equates to approximately eight skipped tags for every join made. This is in contrast to two skipped tags for every 10 joins with the short insert reads.

SOPRA showed a marked difference between Bowtie using -v 0 or -v 3. Although this dataset was the biggest in this study, it is only approximately 90 MB but did seem to push SOPRA to its limits. The SOPRA runs using BWA and Bowtie2 were left running for 12 days before being killed and the run using Bowtie with -v 3 took 10 days. However, the best result using SOPRA was from using Bowtie -v 0, which only took 2 hours to run. MIP crashed on this dataset when using the combined sets of reads mapped with Bowtie2 or BWA.

### Resource requirements

There were large variations in running time between the scaffolders on all datasets (Additional file 1: Figures S6-S17, Additional file 2: Table S3), for which all pre-processing steps were included in addition to the running time of the scaffolding. On each dataset, either SOAPdenovo2 or SSPACE always had the shortest running time and one of SCARPA, MIP or SOPRA was slowest, usually by several orders of magnitude.

The most extreme difference was on the human data using the short and long jump reads combined, where SSPACE’s fastest run (approximately 10 minutes) was over 1,400 times faster than SOPRA’s slowest run (approximately 10 days). However, as noted earlier, SOPRA did complete in 2 hours using different mapping parameters and in fact the faster run produced more accurate results.

Similarly, the differences in run time on the *P. falciparum* dataset were quite extreme. SOAPdenovo2 and SSPACE were the fastest, finishing in under 20 minutes, in contrast to SOPRA which was the slowest to finish successfully, clocking in at around 18 hours. One run of Bambus2 was killed after failing to complete within 12 days. To put these numbers in context, the *de novo* Velvet assembly of the short fragment reads took 44 minutes to compute.

Memory usage was not taken into account when benchmarking the tools because no scaffolder except Bambus2 used an excessive amount of memory in our tests. Of the successful runs, the *P. falciparum* data using all reads had the highest peak memory usage, averaging less than 5 GB. However, Bambus2 had the highest memory usage and was killed after exceeding our memory limit of 30 GB while running on this dataset. SOPRA had the next highest memory usage (13 GB) over all datasets, but this was dependent on how the tool was run. The input reads must be divided into subsets, with the memory usage determined by the size of each subset, so using smaller subsets could reduce the total memory used by SOPRA. MIP had the third highest usage, requiring up to 9 GB on the *P. falciparum* dataset. Typically, the memory required by an assembler to produce contigs for scaffolding is likely to outweigh that needed by the scaffolding tools assessed in this paper. See Additional file 1 for more discussion of the memory usage and running times.

### Wrapper and analysis scripts

We encountered a large variation in the ease of use of the tools in both installation and running. All tools as a minimum need a set of contigs in FASTA format, reads or mapped reads, and insert size information. Some tools require config files, others ask the user to carry out a number of other tasks in order to run the pipeline. For example SOPRA required the reads to be split into chunks, so that it can process the reads one chunk at a time to save excessive memory use. Notably, SSPACE was extremely simple to install and run, which almost certainly contributes to its relatively large number of citations (Table 1) and hence popularity.

All of our scripts are freely available so that the analysis is reproducible and can be run on new scaffolding tools as they appear. In particular, we include wrapper scripts to run each of the scaffolders tested here so eliminating for other users the problems that we encountered with ease of use.

### Lost data

The only scaffolders that lost tags were MIP and SCARPA. Part of the reason for SCARPA losing data could be that in addition to scaffolding together contigs, it also breaks contigs where it thinks there are errors. However, the input contigs contained no errors so it seems unlikely that contig breaking could account for all the lost tags. Our opinion is that that data loss should never happen and should be considered a bug in the software.

### Results summary

On the test cases, SCARPA generated results that were inconsistent with the input data and this trend continued into the real data, where it lost data and was usually outperformed by the other tools. Overall, ABySS, Opera and MIP tended to perform reasonably well on real data, but usually did not produce the highest quality results. MIP also lost data. This agreed with the test cases, where Opera did not follow the majority rule and although MIP did so, it also produced some unpredictable results when there was no clear solution to the scaffolding problem.

SGA always chose the solution with the most evidence in the test cases and, although it chose randomly between equally likely solutions, was still very conservative on the real data and had a low error rate, suggesting that choices with equal evidence may actually occur relatively infrequently. Bambus2 was similar to SSPACE on the test datasets, but on the real data its accuracy was more variable. SOPRA and SSPACE generally performed well, agreeing with their predictable test case output. SOAPdenovo2 made very few joins in all of the tests, which is at odds with the real data, where it tended to make more joins than any other scaffolder usually with an acceptable error rate.

## Discussion

The ultimate aim of the scaffolding process is to join assembled contigs into longer sequences. The outcome depends on the quality of the input contigs, the size and quality of read pair libraries and the structure of the genome. To dissect these problems we first generated an apparently trivial test set (Figure 1, Additional file 1: Figures S1-S3) and were surprised by some of the unexpected or incorrect scaffolds that were produced. In particular, SOAPdenovo2 made few joins, in contrast to its aggressive behaviour on larger datasets. SCARPA tended to make either no joins or many errors and SGA joined more contigs than we expected given its apparent conservative nature on larger datasets.

We found it necessary to write wrapper scripts for most of the tools in order to use them in an automated fashion. These scripts have been made available together with all our analysis scripts. We would prefer all the tools to clearly separate off the read mapping stage and allow the scaffolding step to accept a BAM or SAM file as input, permitting any mapper to be used. This is already possible for ABySS, Bambus2, MIP, SCARPA, SGA and SOPRA. A default mapping stage or a recommended mapper and its parameters could still be included for ease of use.

The scaffolding process is critically dependent on the correct mapping of the reads to contigs. However, different mappers and parameters can produce vastly different mapping results, as seen in the human large insert reads where the percentage of reverse reads mapped ranged from 6% to 85% (Table 2). Mapping low quality reads becomes a balance between allowing enough mismatches so that the reads map, but too many mismatches results in false read placement and scaffolding errors. Accuracy of read mapping is more important than aiming for as many reads mapped as possible. To this end, we recommend Bowtie2 as the best mapper for use with SGA and Bowtie for use with SOPRA. Bowtie with the option -v 0, which is the default for SSPACE, should be used for the remaining tools that let the user control the mapping. Users may wish to tailor their mapping based on knowledge of their genome of interest, for example BWA was not a good choice for SGA on *P. falciparum.*

Generally, the software performed very well on simulated data, with many runs producing perfect scaffolds. The difficulties arose when real libraries and genomes were used. Although the effectiveness of the scaffolders differed depending on the input data, some broad recommendations can be made. Overall, SGA is the most conservative tool and should be the one of choice if minimising errors is most important to the end user. However, this comes at a cost of making significantly fewer joins than other tools. SOPRA appears to strike the best balance between aggressively making joins, with a reasonably low error rate. It also performed the best when given two sets of reads from different fragment size libraries, presumably because it combines the linking information from both libraries into its graph. The downside is its scalability, with the run times becoming impractically long on large datasets. It is worth reiterating that SOPRA was the first stand-alone scaffolder tool developed for NGS data at a time when datasets were significantly smaller than they are now, so that scalability issues should come as no surprise.

SSPACE and SOAPdenovo2 are good choices if a short running time is important since they were the fastest to run on all datasets but still performed well with respect to correct *versus* incorrect joins. SSPACE tended to be more conservative than SOAPdenovo2 on most of our datasets. Interestingly, SSPACE comfortably has the most citations of any of the scaffolding tools and is also the easiest of the tools to install and run. We do not recommend using MIP, OPERA or SCARPA because SGA, SOPRA, SOAPdenovo2 and SSPACE generally outperformed them.

Further improvements for scaffolding could come from four approaches: higher quality libraries, improvement of the mapping and the contigs to be scaffolded, improvement of the scaffolding algorithm itself or the use of new methods that have recently been developed that utilise chromatin interactions [24], single cell sequencing [25] or optical mapping [26] to orientate and order contigs. Obviously, if the insert size of the library is smaller than repetitive elements in the genome or the sequencing reads do not map uniquely and are prone to errors, there will be a general limit to what a scaffolder can achieve. Longer read lengths and also new techniques such as those mentioned above will help to improve results.

To overcome some of these problems, especially noise generated from incorrect read mapping, the following steps could be used: (1) correct the reads before mapping; (2) correct the reference for errors with REAPR [27], or base errors that make mapping difficult with iCORN2 [28]; (3) the contigs ends could be extended by walking, as possible in SSPACE; (4) if enough coverage exists, the mapping could be filtered to remove spuriously mapped reads or even reads with at least one mis-match; (5) mate pairs could have failed pairs (where circulation failed during library construction, resulting in paired end reads from a short fragment) filtered by removing reads mapped within a short distance of contig ends; and (6) scaffold iteratively, starting with a high number read pairs required to join contigs and progressively reduce the stringency.

Improvements to the tools are difficult, due to the complexity of the problem, but they could test each join with assembly accuracy metrics such as those of REAPR. Also, tools could improve their handling of heterozygosity, as can be seen from the results of the test cases. However, handling of polyploid genomes is an assembly problem that is not restricted to scaffolding and current assemblers generally assume a haploid genome. The amount of contig skipping, particularly prevalent from large insert libraries, could be reduced by removing short contigs before scaffolding, then filling the gaps in scaffolds with tools such as GapFiller [29] or IMAGE [30].

## Conclusions

The scaffolding problem of joining contigs using read pairs is simple to understand, but it is computationally difficult and can only be solved approximately. We recommend the use of SGA, SOPRA, SOAPdenovo2 or SSPACE, depending on the requirements of the output and the size of the genome. No tool identified more than 90% of the joins across any of the genome-scale datasets, typically leaving the *P. falciparum* and human assemblies in thousands of scaffolds. Although the need for better quality large insert size libraries is evident, there is still scope for improvements to existing scaffolding algorithms.

## Materials and methods

### Small test cases

Contigs of random sequence of length 5 kb were generated, where each of A, C, G and T had an equal probability of being chosen. The contigs were arranged in a number of configurations (see Additional file 1: Figures S1-S3) and corresponding perfect Illumina read pairs were generated, with fragments sampled uniformly from the contigs, and fragment lengths sampled from a normal distribution with mean 500 and standard deviation 30. The reads all had length 76 bp and every base was given a quality score of 40. We then trimmed 40 bp off the end of each contig and used these with the simulated reads as input to each scaffolder. Figure 1a shows an example of this read and contig generation and the corresponding graph is in Figure 1b. Since some scaffolders use heuristics, each test was generated and run five times.

For consistency, the contigs were always put in alphabetical order of their names within the FASTA file input to the scaffolders. We checked that none of the contigs had any sequence in common by running BLAST against themselves (with blastall version 2.2.25 settings -p blastn -e1e-10), which reported no hits other than each contig to itself. The reads were mapped with Bowtie2 for SGA, SOAP2 for SOAPdenovo2 and using Bowtie -v 0 for all other scaffolders.

### Generating input data to scaffolders

A set of contigs output from an assembler served as the starting point when producing input contigs for scaffolding. For each of the GAGE datasets, we used contigs from the Velvet assemblies. A *de novo* assembly of *P. falciparum* 3D7 was generated from Illumina paired end reads with a fragment size of 625 bp (ENA accession number ERR034295) using Velvet version 1.2.07 with a kmer of 55 and options -scaffolding no -ins_length 625 -exp_cov auto -cov_cutoff auto. Two sets of perfect contigs were made from the *S. aureus* genome (excluding the plasmid sequences), with lengths of 3 kb and 10 kb separated by gaps of 50 bp and 300 bp, respectively (see Figure 2a).

The following process was used to generate contigs with no errors and sequence tags for tracking after scaffolding (see Additional file 1: Figure S5). The contigs from an assembler were aligned to the reference genome with the Nucmer package of MUMmer [31] version 3.23, using the options -l 180 -i 98 -q to delta-filter. Then for each contig, all overlapping hits in the reference were merged and the resulting list of positions was used to make new contigs using the sequence from the reference (Figure 2b). In this way, each assembly contig gave rise to one or more artificial contigs. The result was contigs that are similar to the output of an assembler, but contain no mis-assemblies.

Next, we looked for a sequence tag within each contig that could uniquely identify that contig. For a given contig, we began by mapping its middle 50 bases to all the contigs and to the reference sequence using Bowtie2 version 2.0.5 with the default settings. If it had exactly one perfect hit to the contigs (that is, its contig of origin) and to the reference sequence, then we kept that tag. Otherwise, the process was repeated with tags of length 100, 200, 400, 600, 1,000, 2,000, and 5,000, stopping when a unique tag was found. The end result was a set of contigs containing no errors that could be unambiguously tracked before and after scaffolding. These were used as input to the scaffolders, together with paired reads.

All ‘short jump’ reads for the GAGE datasets were used, which have insert sizes of approximately 3 kb. We also used the ‘long jump’ library, with insert length 35 kb, from the human chromosome 14 dataset. For *P. falciparum*, the reads that made the assembly and a second set of Illumina reads (ENA accession numbers ERR163027-9) with an insert size of 2.7 kb were used. Perfect read pairs at 30× coverage were generated by sampling fragments uniformly from the *S. aureus* reference sequence, with fragment lengths sampled from a normal distribution. We used fragment lengths of 500 bp and 3,000 bp, with standard deviations of 30 and 200, respectively, taking the end 76 bases from each fragment to make read pairs. Every base in the simulated reads was given a quality score of 40.

### Read mapping

Opera, SOAPdenovo2 and SSPACE take the reads as input and run the mapping for the user. Opera lets the user choose between Bowtie and BWA, with no option to change any mapping parameters. It runs BWA with default settings and Bowtie with -m 1 (only report reads that have exactly one hit) and -v 3 (allow up to three mismatches in alignments). SSPACE uses Bowtie with the option -m 1 -v 0, but does allow the user to change the value of -v. SOAPdenovo2 uses its own read mapper.

MIP, SCARPA, SOPRA and SGA need the user to map the reads. SGA requires reads mapped as paired reads, whereas the others need the mapping performed as if the reads were not paired. We used BWA v0.7.4, Bowtie v0.12.8 (always with option -m 1 and the either -v 0 or -v 3 for consistency with Opera and SSPACE) and Bowtie2 v2.0.5 to make input to MIP, SCARPA, SOPRA. For SGA we only used BWA and Bowtie2 because Bowtie does not report reads within a pair that map to different contigs and so yields no scaffolding information. Except for the options noted above for Bowtie, the only mapping settings changed were those that set the maximum insert size (-a for BWA, -X for Bowtie and Bowtie2, the values are in Additional file 2: Table S4) to produce BAM files for SGA. The long jump human reads were found to be of low quality or have large insertions near their start, so before mapping we trimmed the first 19 bases off each read.

### Scaffolding

All scaffolders were run using their default settings except for the following exceptions. SSPACE was run using its default settings and also using -g 3 (which passes -v 3 to the Bowtie mapping call). We changed options to prevent tools from running in multi-threaded mode so that running times could be compared between scaffolders. In particular, we changed the source code of Opera so that the Bowtie call used one thread (Bowtie’s default) instead of the hard-coded five threads. For SOAPdenovo2 we set the number of threads to one with -p 1. The parameters used, such as insert size, for each dataset are given in Additional file 2: Table S4.

The scaffolders varied in the amount of work needed to prepare data in a suitable form for input and in the number of stages required to get from reads and contigs to a final set of scaffolds. The MIP scaffolder requires a ‘coverage file’ that contains the read depth of each contig. This was generated from a BAM file using a custom Perl script, which is available together with all the wrapper scripts made to make each tool simple to run.

The *P. falciparum* and human datasets each had two sets of reads of different insert sizes. We ran each scaffolder separately on both sets of reads, and then a third run of each scaffolder with all reads. Some incorporate information from both sets into the scaffolding graph simultaneously, whereas others simply scaffolding using the libraries sequentially, starting with the smallest insert size.

### Evaluation

The output of each scaffolding tool is a set of scaffolds, which was evaluated by identifying the position of each unique sequence tag that was generated for each input contig. The positions were determined by mapping the tag sequences back to the scaffolds using Bowtie2. There are several possible cases when analysing the location of these tags (Figure 2c). A join between two contigs was classified as correct if the corresponding tags were in the correct orientation and distance from each other. We counted the distance as correct if the error in distance was less than the fragment length. If tags were in the wrong orientation, from different chromosomes or the wrong distance apart then the join was counted as incorrect. It is possible that a join could be correct, but have skipped some sequence (see Figure 2c, tags 2 to 4). We counted these cases separately from incorrect joins, calling them ‘skipped tags’, since the scaffolder could have done better by recognising that other contigs could have been inserted between the two joined contigs. A final possibility is that a tag is not found at all in the output scaffolds, which we call a ‘lost’ tag and consider being a serious error because a tool should not lose any data.

In order to summarise the wealth of data produced from many scaffolding runs over 12 combinations of contigs and reads, we gave precedence to five key metrics: the number of correct joins, incorrect joins, skipped tags, lost tags and total running time. All pre-processing stages were included in the running time, such as mapping reads before running a scaffolder (see Additional file 1 for more details).

A distribution of summary scores for each tool on each dataset was obtained by weighting the metrics with a range of values, as follows. Correct joins: 10, 20, 40, 80, 160; bad joins: 10, 20, 40, 80, 160; lost tags: 20, 40, 80, 160, skipped tags: 10, 20, 40, 80, 160; total CPU: 1, 2, 3, 4, 5. To emphasise the importance of accuracy, we only kept combinations of weights where: (1) bad joins ≥ correct joins, (2) bad joins ≥ 2 * skipped tags, (3) lost tags ≥ correct joins. Redundant combinations were only counted once, for example doubling all weights produces the same final score.

## Competing interests

The authors declare that they have no competing interests.

## Authors’ contributions

All authors conceived the project and wrote the manuscript. MH wrote all scripts. MH and TDO carried out analysis of output from the scaffolders. All authors read and approved the final manuscript.

## Supplementary Material

Additional file 1 Extra details of the methods, results of all test cases and figures displaying the results of all datasets. Click here for file

Additional file 2 Supplementary tables. Click here for file

## Data Availability

The ENA accession numbers of the *P. falciparum* reads are ERR034295 and ERR163027-9 and the reference genome can be downloaded from ftp://ftp.sanger.ac.uk/pub/pathogens/Plasmodium/falciparum/3D7/3D7.latest_version/version3/Pf3D7_v3.fasta.gz. All wrapper and analysis scripts are freely available from https://github.com/martinghunt/Scaffolder-evaluation. The simulated data can be generated using those scripts. The remaining data were all from the GAGE project and can be downloaded from http://gage.cbcb.umd.edu/data/index.html.
