# Supplementary material for: A comprehensive evaluation of assembly scaffolding tools
Source: Genome Biol. 2014 Mar 3;15(3):R42. doi: 10.1186/gb-2014-15-3-r42 (PMC4053845; doi:10.1186/gb-2014-15-3-r42)
Supplement: Additional file 1 — Extra details of the methods, results of all test cases and figures displaying the results of all datasets. [file gb-2014-15-3-r42-S1.pdf]

Supplementary Material of:

A comprehensive evaluation of assembly scaffolding tools

Martin Hunt[1], Chris Newbold[2,1], Matthew Berriman[1], Thomas D. Otto[1]

[1]Parasite Genomics, Wellcome Trust Sanger Institute, Wellcome Trust Genome Campus, Cambridge, CB10 1SA, UK

[2]Weatherall Institute of Molecular Medicine, University of Oxford, John Radcliffe Hospital, Oxford, OX3 9DS, UK

Running times and memory

The running times and memory usage are given in Supplementary Table 3 and visualised in Supplementary Figures 6–17.

For tools where the user is required to manually run the read mapping, an in-house mapping pipeline was used. This comprised of the following stages.

1. Map the reads to produce a BAM file.
2. Sort the BAM file by coordinate using samtools sort.
3. Mark duplicates using Picard.

The peak memory usage was separated out into memory used by the mapping pipeline and memory used by the actual scaffolding stage. For the small datasets, the scaffolding uses little memory but the memory used by Java is dependent on how it is run and could likely be reduced (for example, using `-Xmx`).

Classifying tag pairs

When identifying the correct and incorrect joins made by each scaffolder there were several possibilities. After mapping the tags to the scaffolds, each pair of consecutively mapped tags within a scaffold was analysed and a bitwise flag used to store the information for that pair. The flags are reported in columns I to N in Supplementary 3 and have the following meaning:

0 – correct pair of tags.

1 – tags originate from same reference sequence, but their orientation in the scaffolds is incorrect.

2 – tags originate from different reference sequences.

4 – tags originate from the same reference sequence but are the wrong distance apart.

8 – tags originate from the same reference sequence but are not in the correct order.

A flag of 5 means that $4 + 1$ happened, i.e. a pair of tags that originated from the same reference sequence, but their orientation and order were incorrect. Similarly, $12 = 8 + 4$ means that two tags were from the same reference sequence, but were the wrong distance apart and in the wrong order.

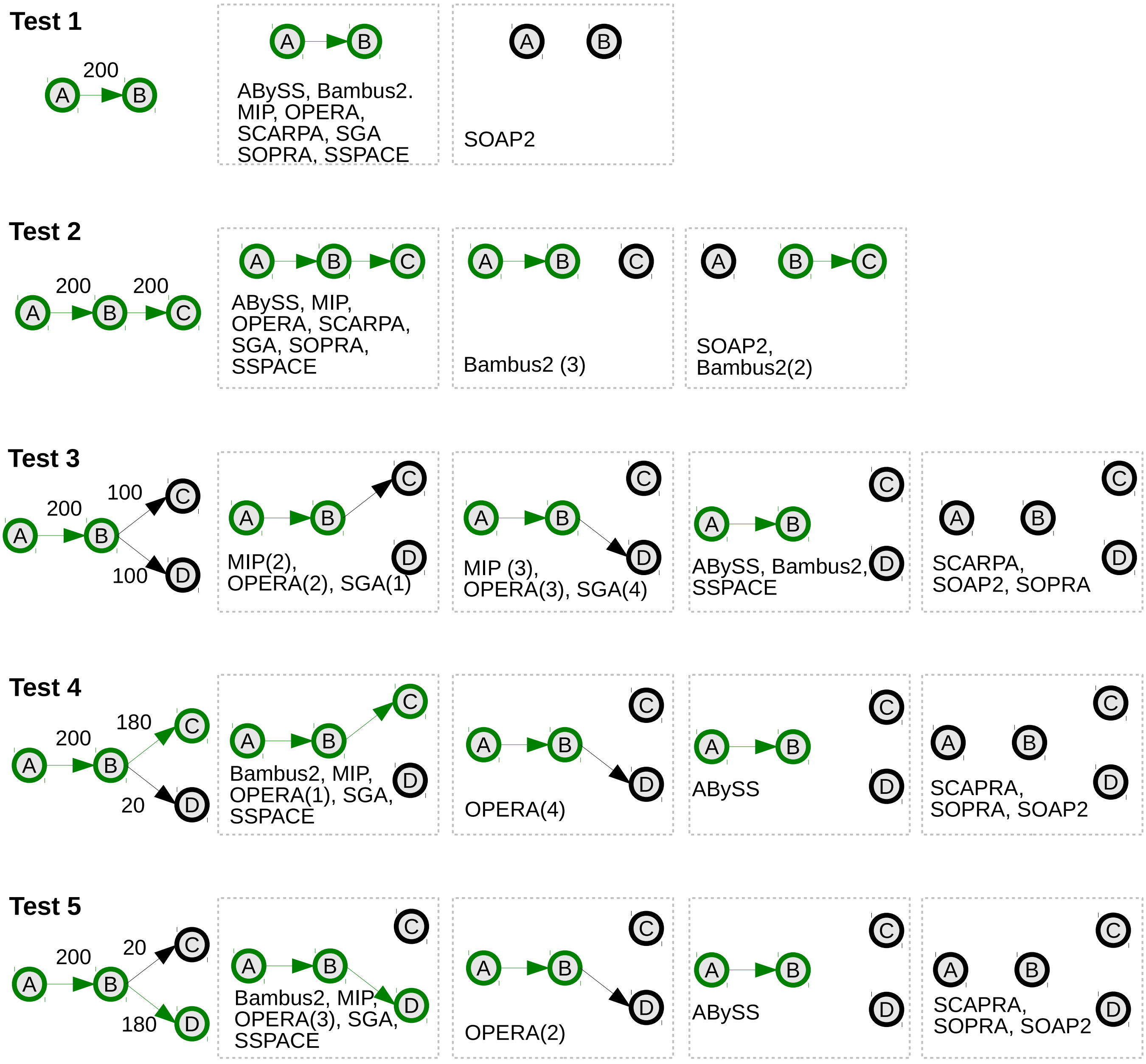

Figure 1: Test cases 1 to 5. See the legend of main Figure 1B for more details.

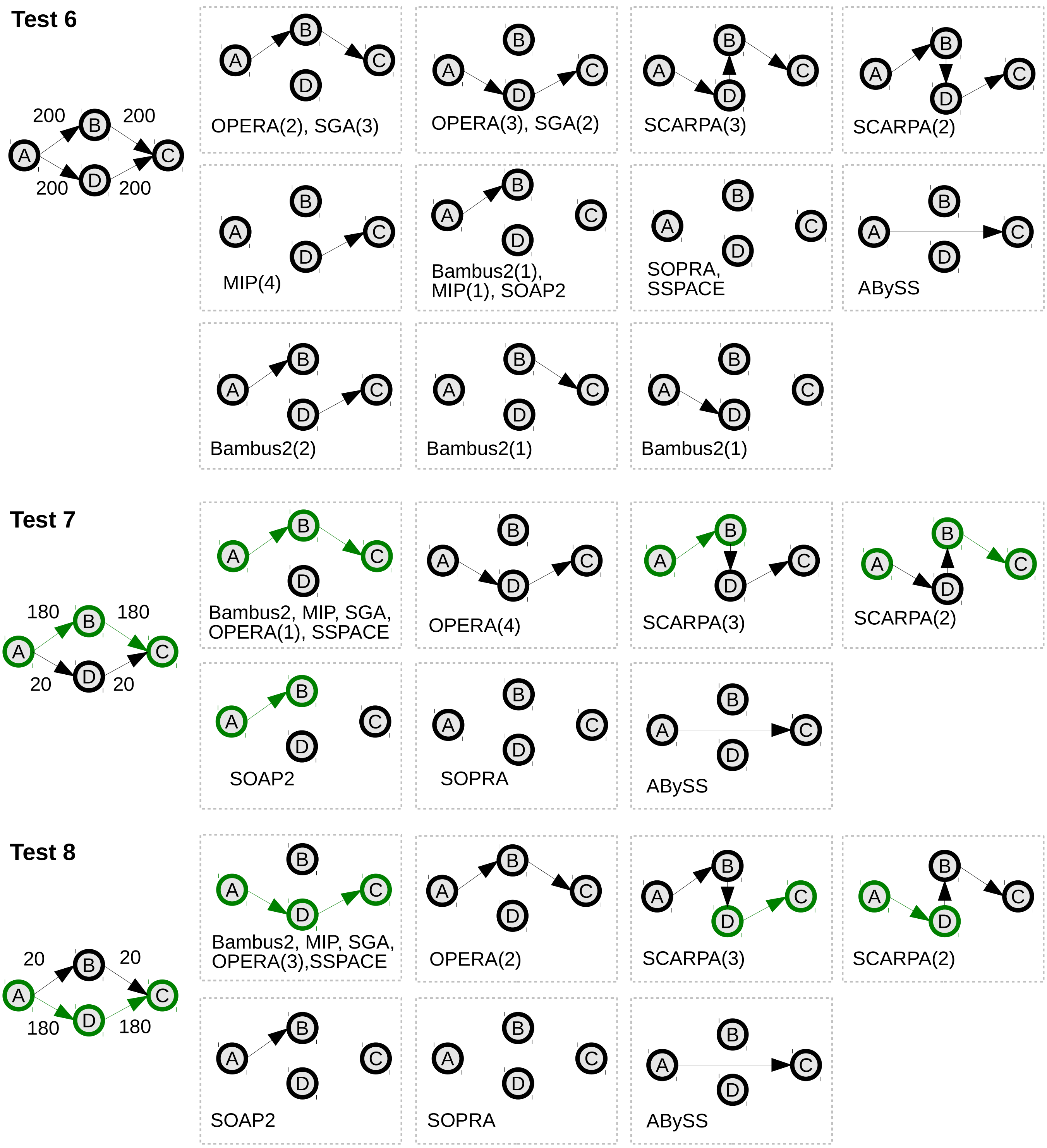

Figure 2: Test cases 6 to 8. See the legend of main Figure 1B for more details.

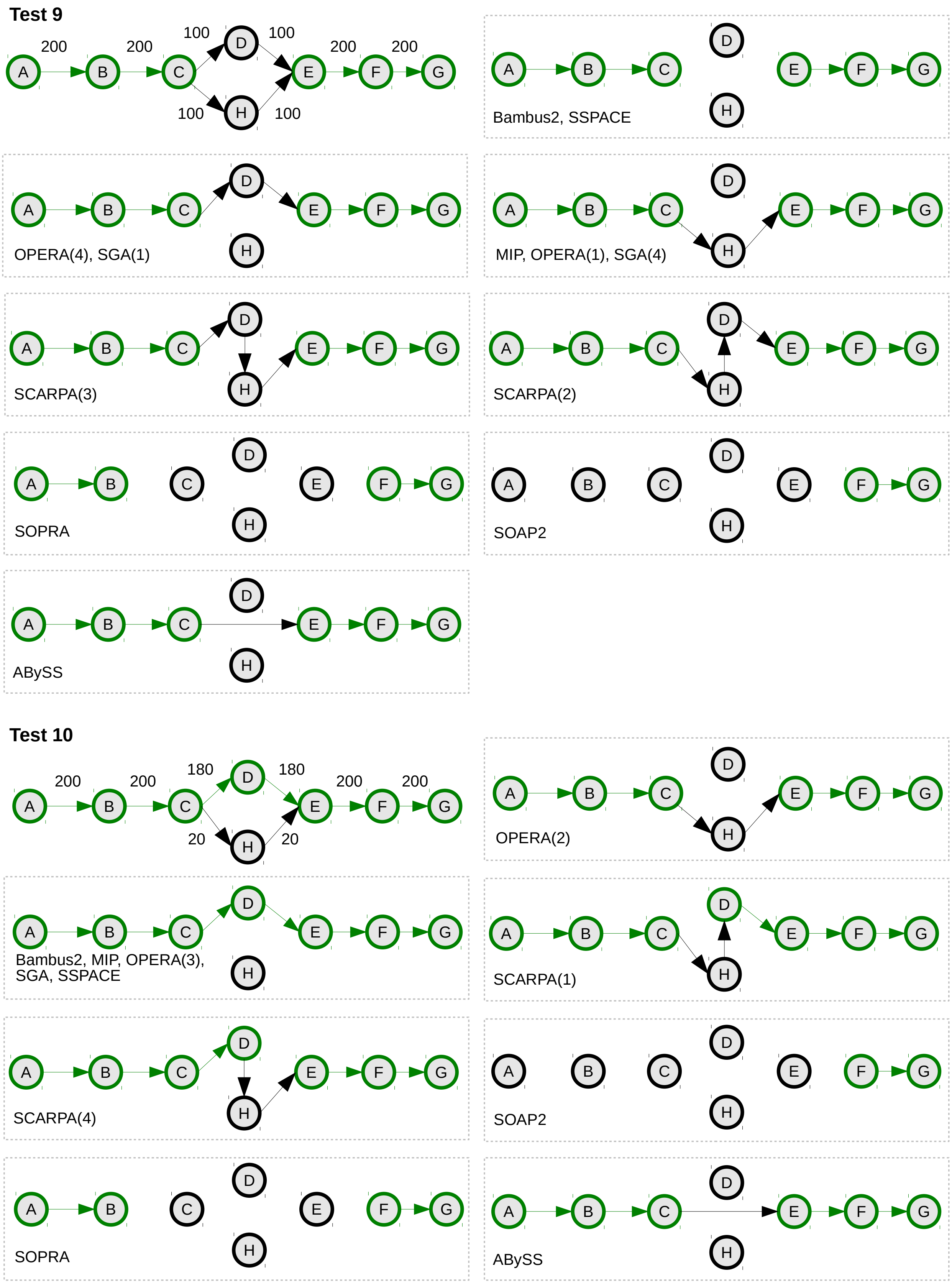

Figure 3: Test cases 9 and 10. See the legend of main Figure 1B for more details.

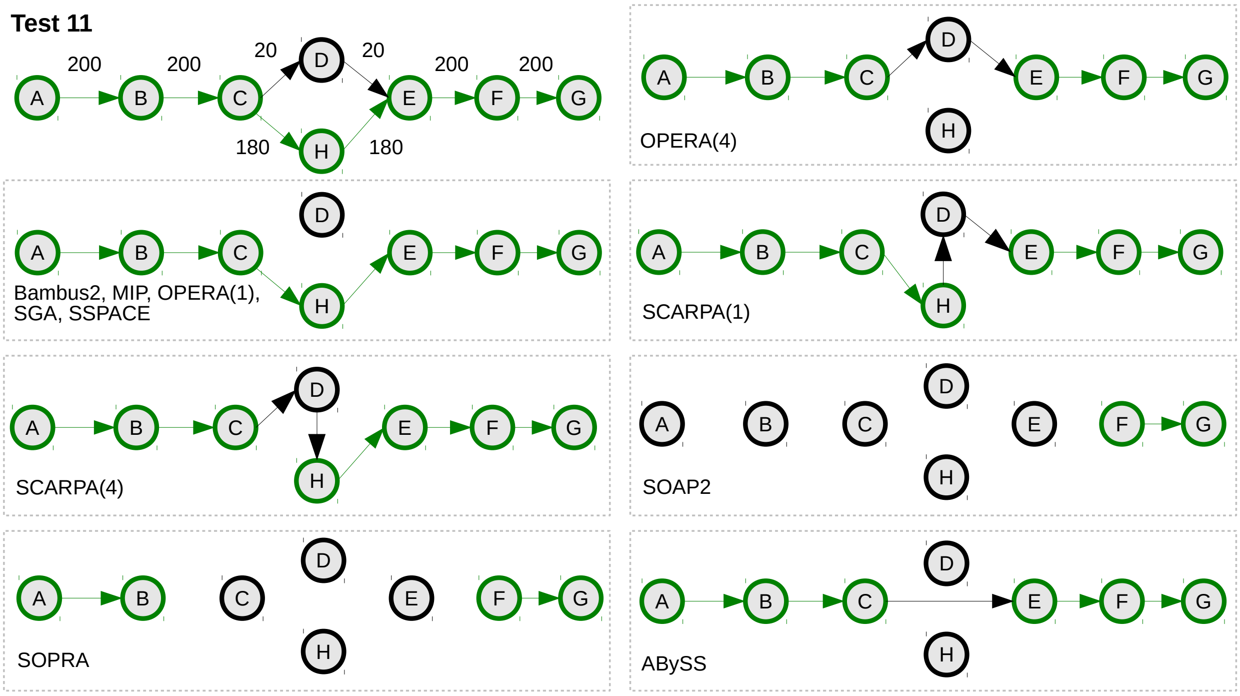

Figure 4: Test cases 11. See the legend of main Figure 1B for more details.

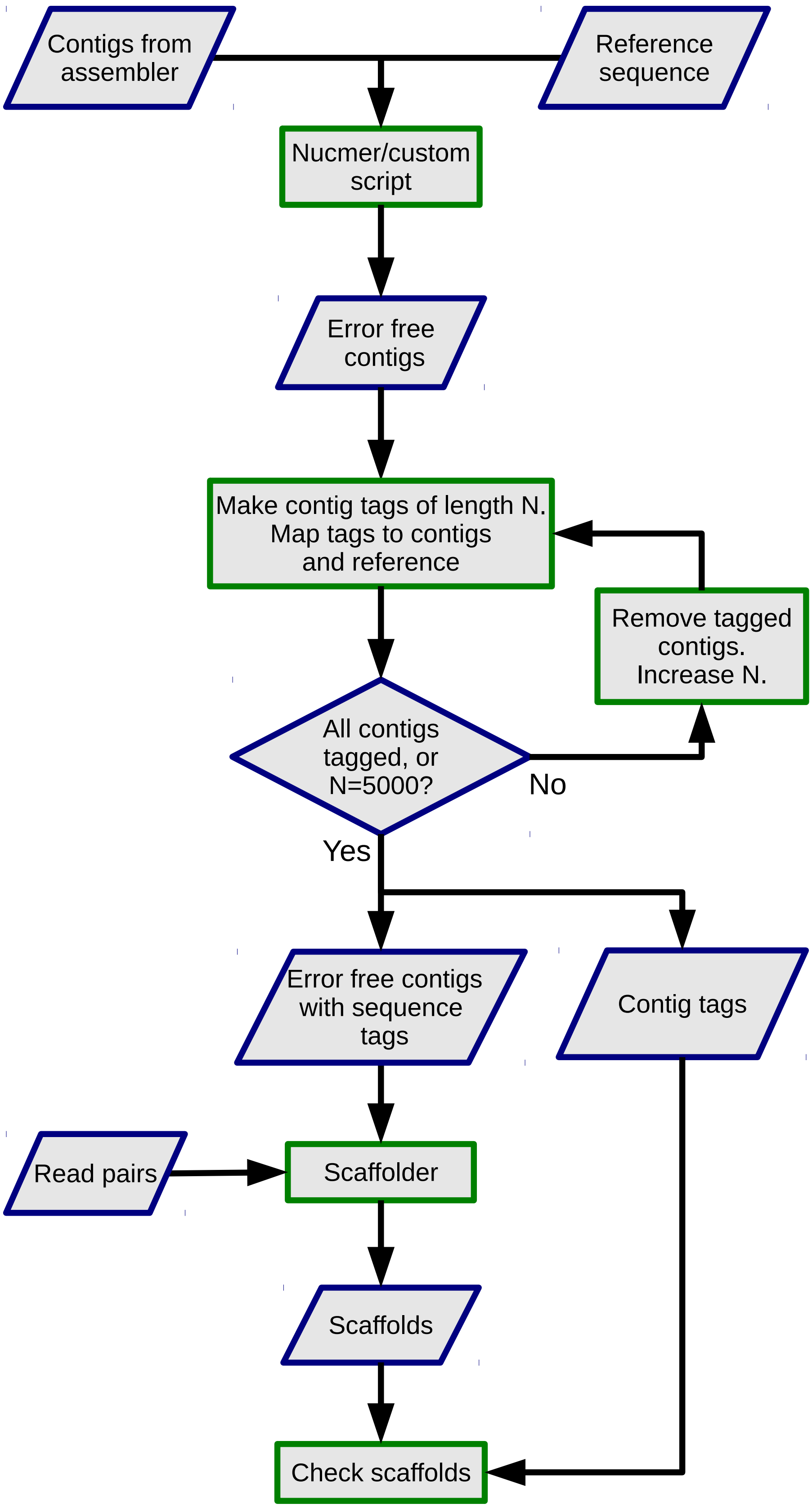

Figure 5: Flowchart of methods used to generate artificial contigs and tags.

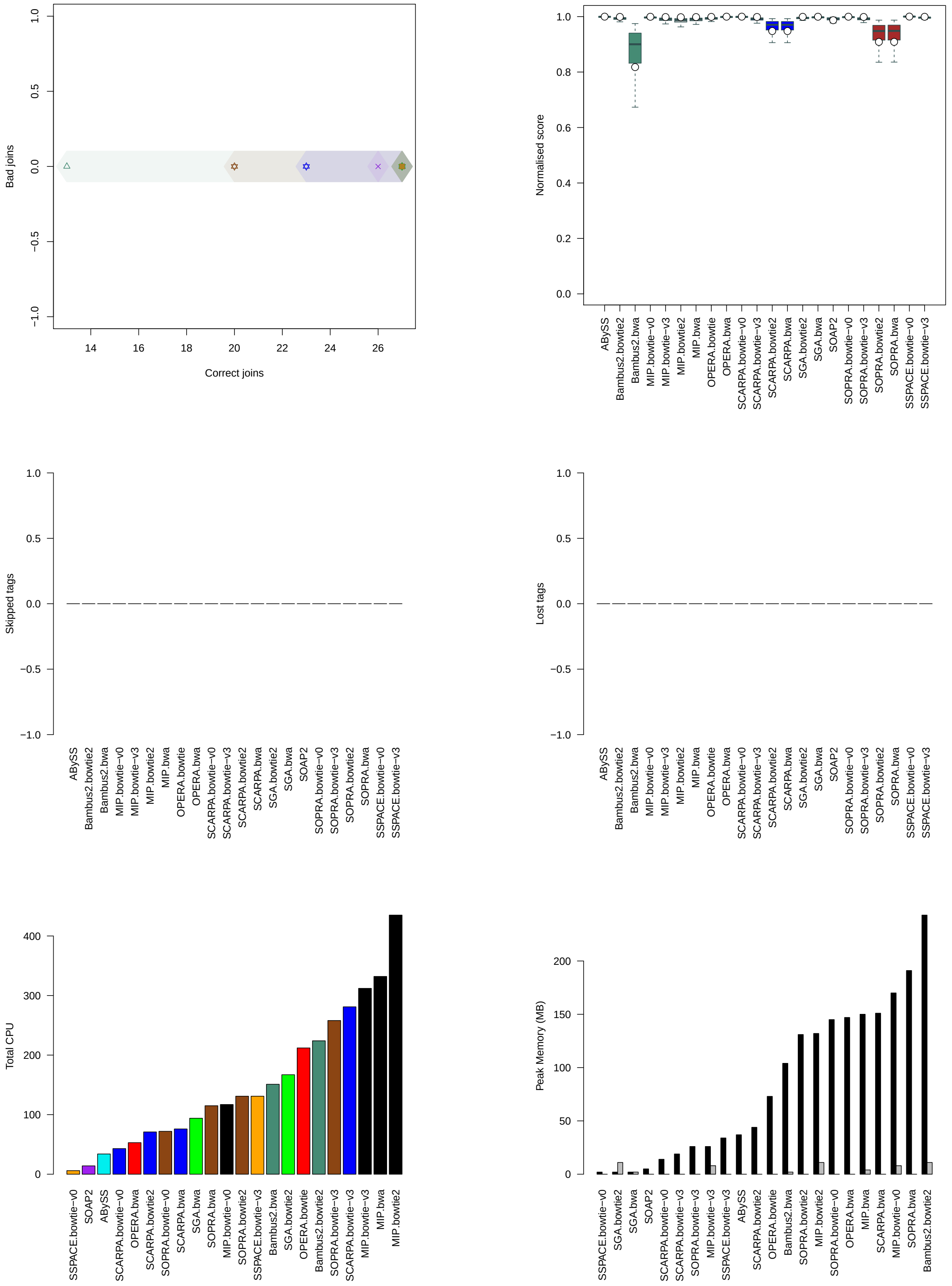

Figure 6: Results of scaffolding on the *S. aureus* simulated dataset, with 10kb long contigs and short fragment reads

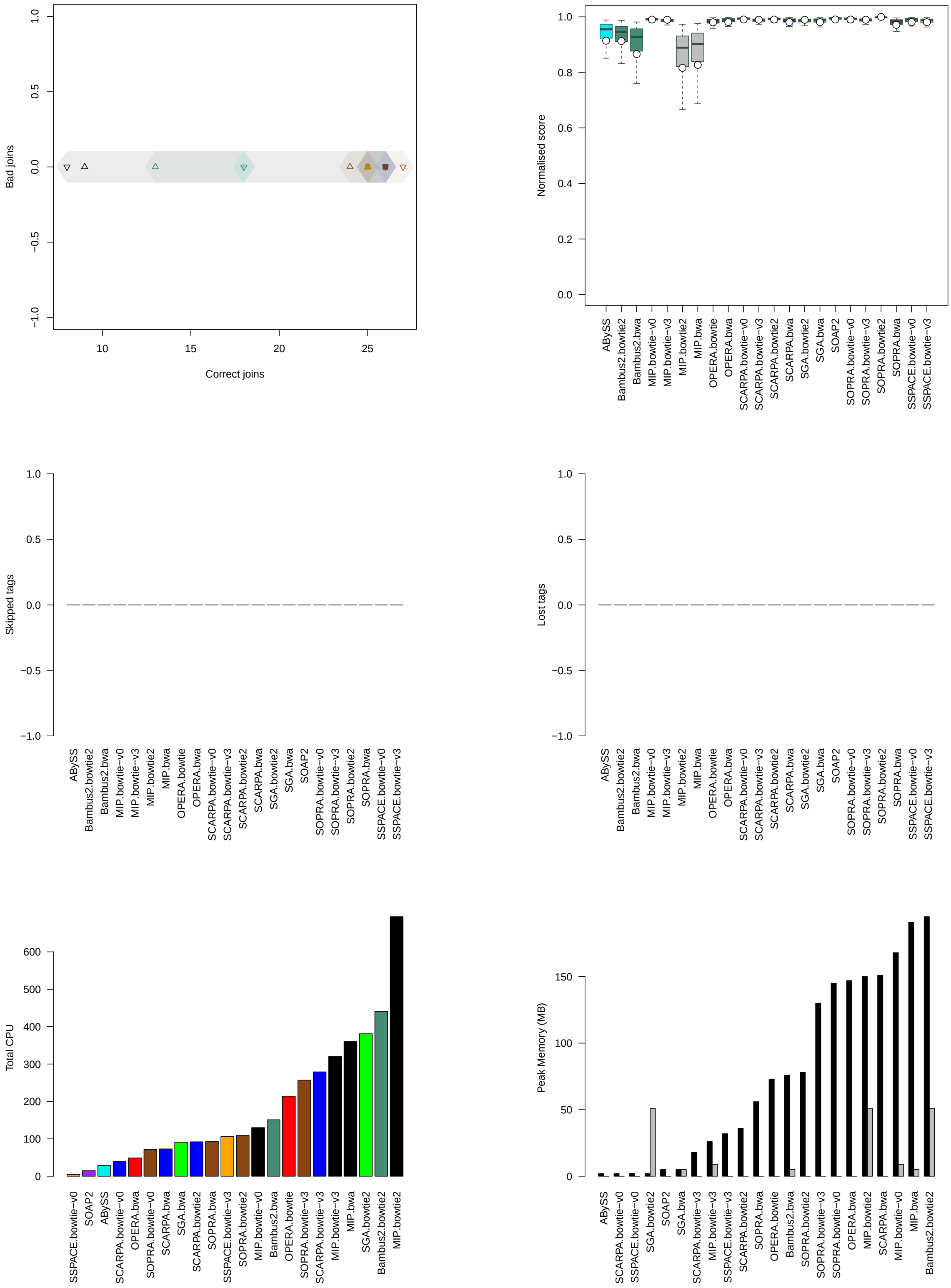

Figure 7: Results of scaffolding on the *S. aureus* simulated dataset, with 10kb long contigs and long fragment reads. See the legend of Supplementary 6 for an explanation.

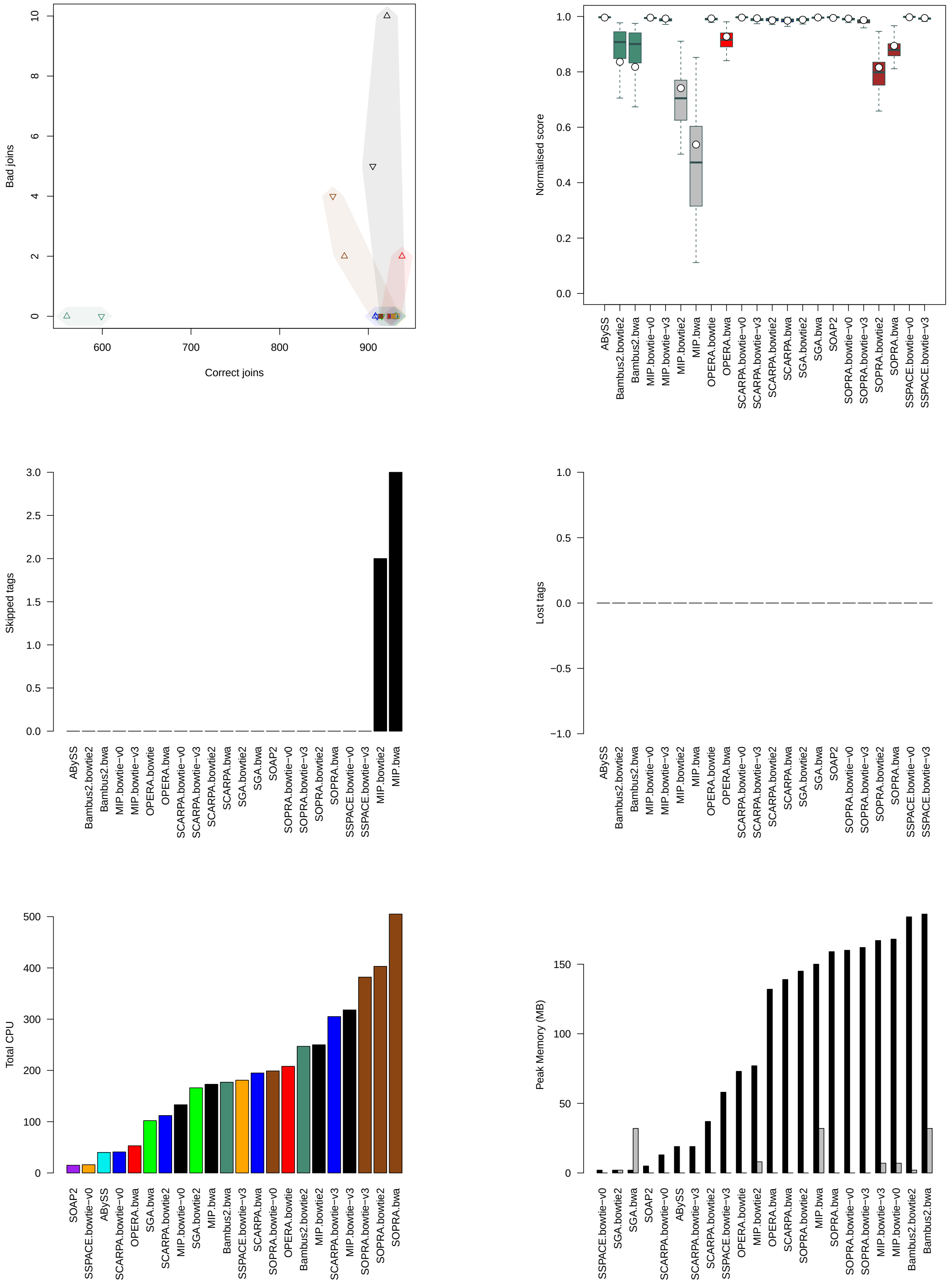

Figure 8: Results of scaffolding on the *S. aureus* simulated dataset, with 3kb long contigs and short fragment reads. See the legend of Supplementary 6 for an explanation.

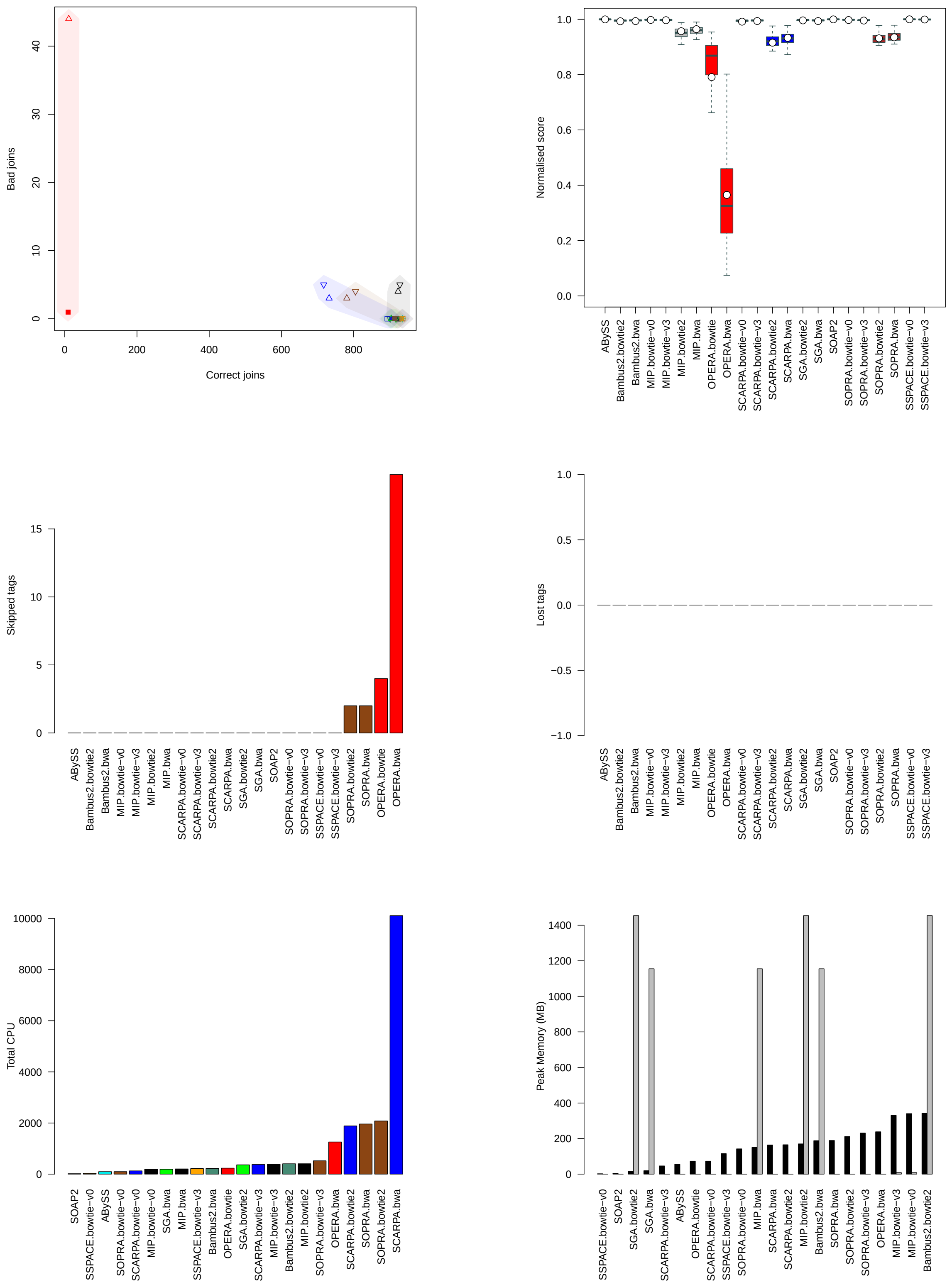

Figure 9: Results of scaffolding on the *S. aureus* simulated dataset, with 3kb long contigs and long fragment reads. See the legend of Supplementary 6 for an explanation.

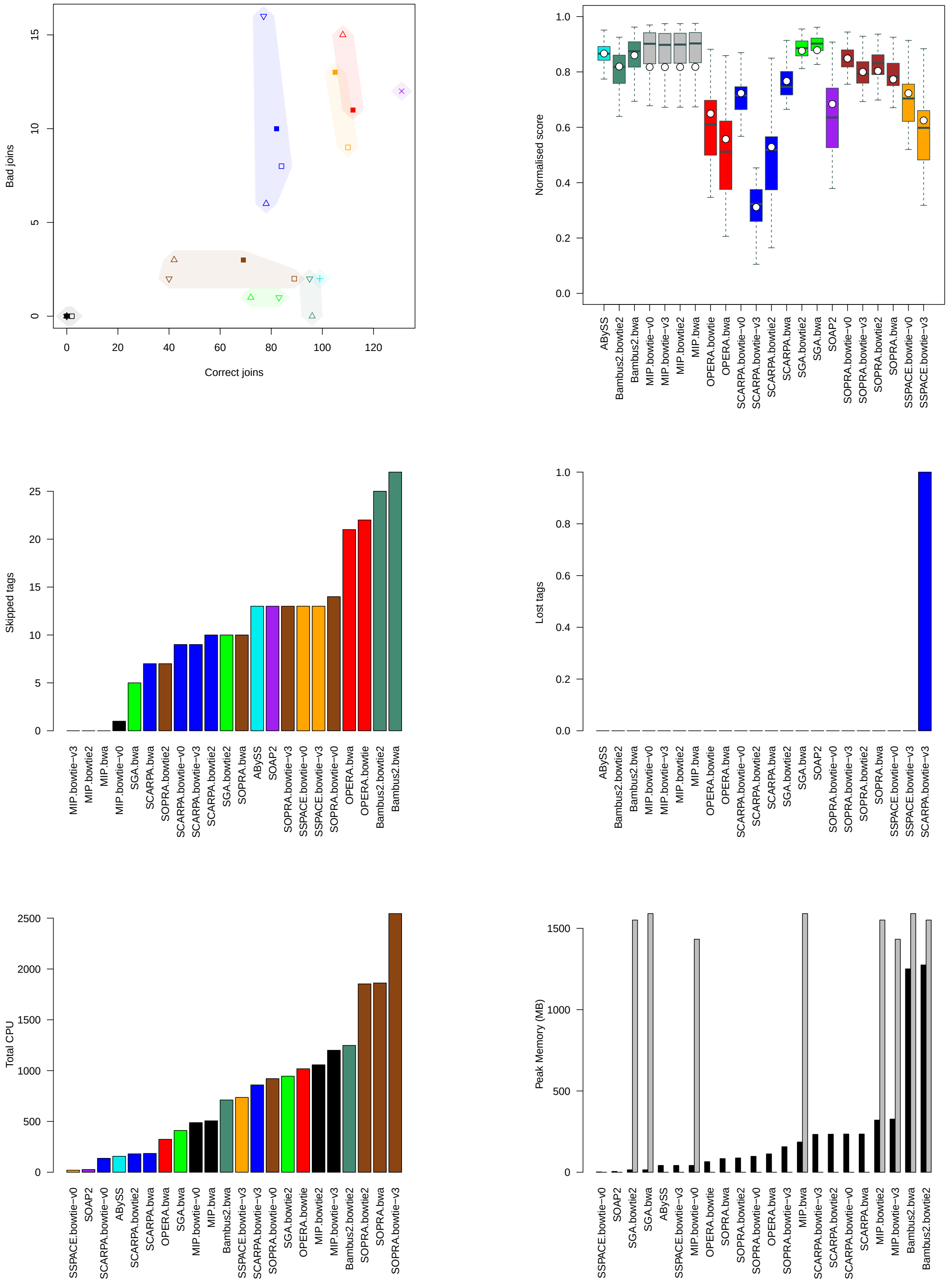

Figure 10: Results of scaffolding on the *S. aureus* GAGE Velvet contigs. See the legend of Supplementary 6 for an explanation.

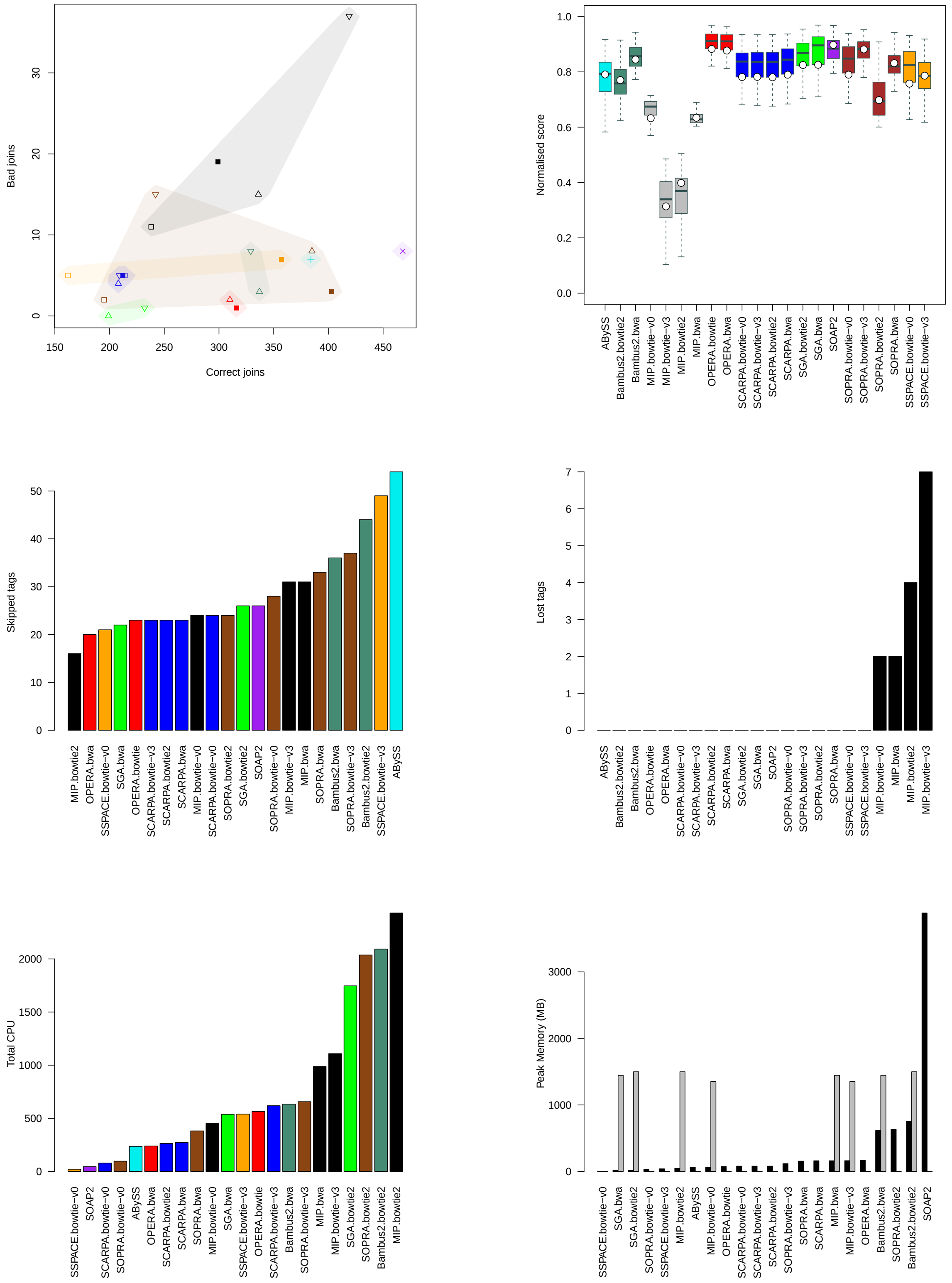

Figure 11: Results of scaffolding on the *R. sphaeroides* GAGE Velvet contigs. See the legend of Supplementary 6 for an explanation.

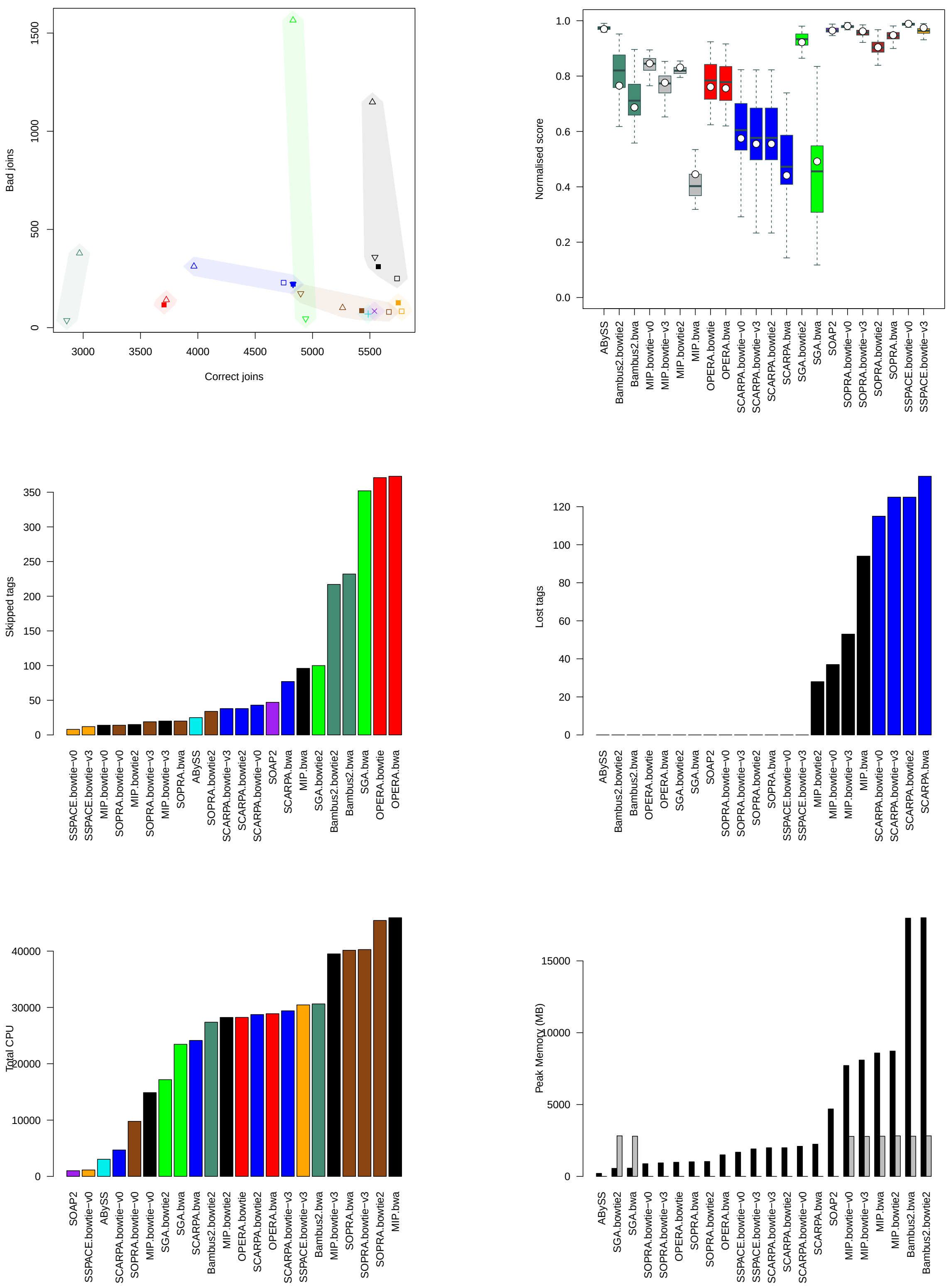

Figure 12: Results of scaffolding on the *P. falciparum* contigs using short fragment reads. See the legend of Supplementary 6 for an explanation.

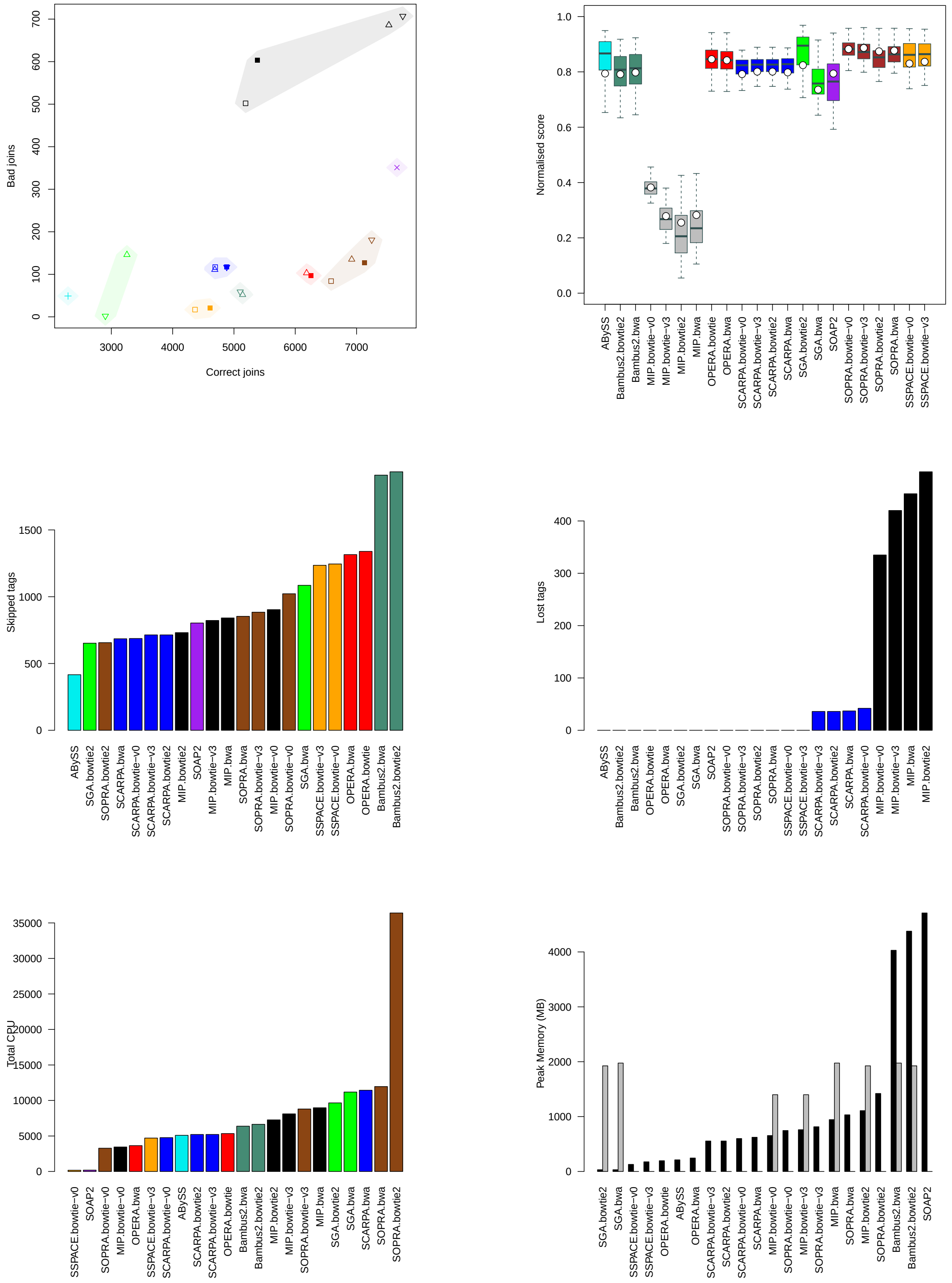

Figure 13: Results of scaffolding on the *P. falciparum* contigs using long fragment reads. See the legend of Supplementary 6 for an explanation.

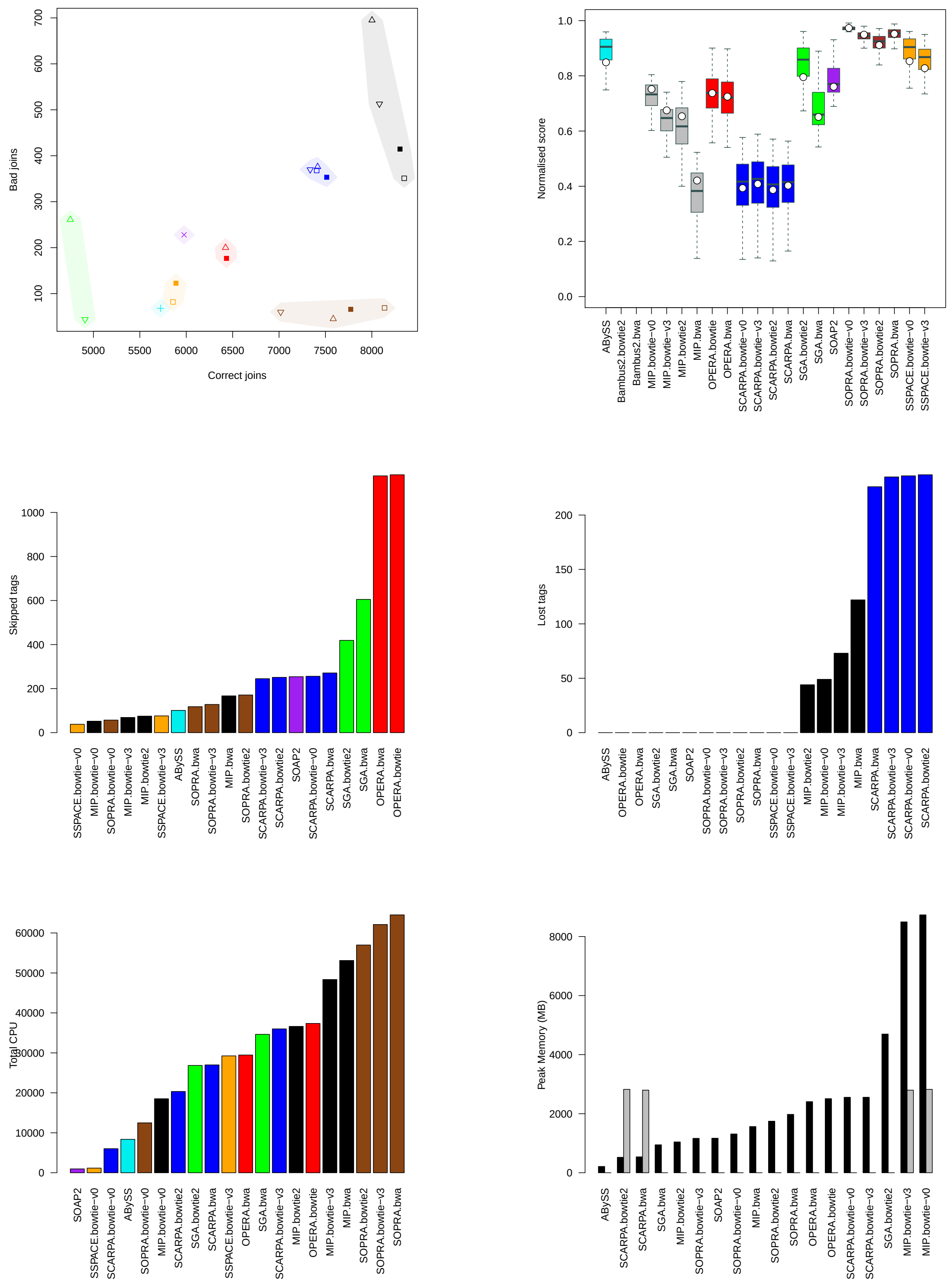

Figure 14: Results of scaffolding on the *P. falciparum* contigs using short and long fragment reads. See the legend of Supplementary 6 for an explanation.

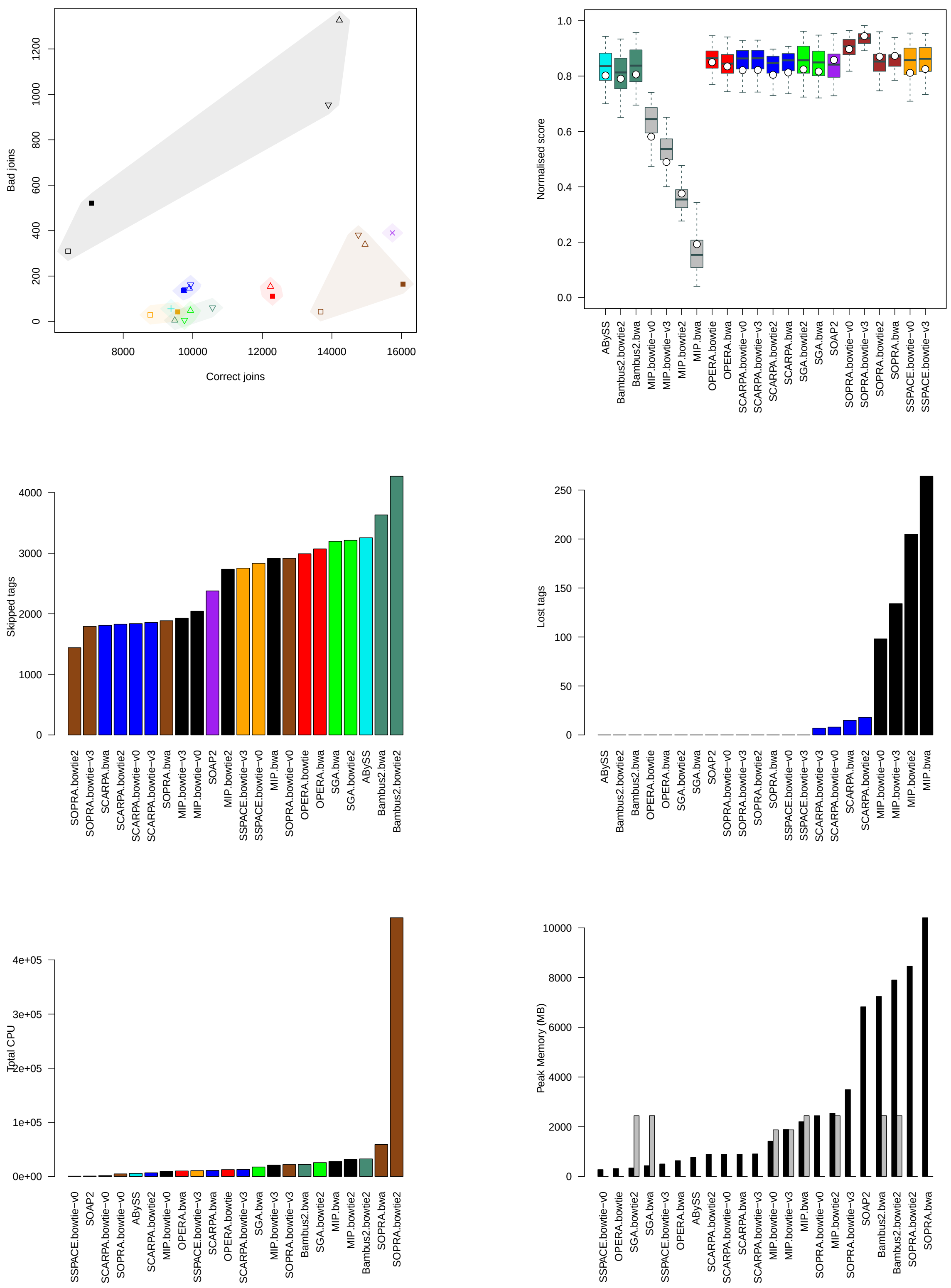

Figure 15: Results of scaffolding on the human chromosome 14 GAGE Velvet contigs using short fragment reads. See the legend of Supplementary 6 for an explanation.

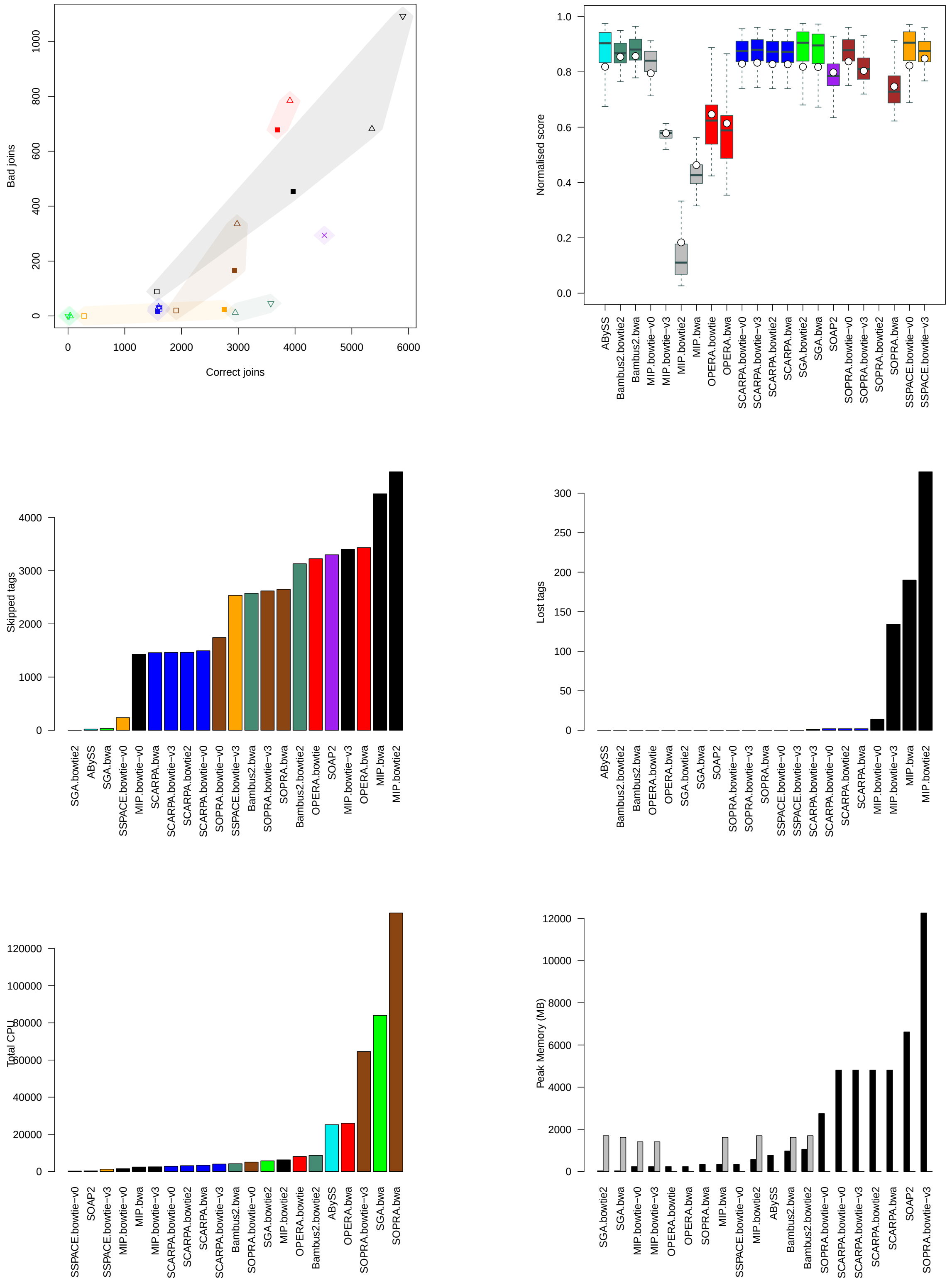

Figure 16: Results of scaffolding on the human chromosome 14 GAGE Velvet contigs using long fragment reads. See the legend of Supplementary 6 for an explanation.

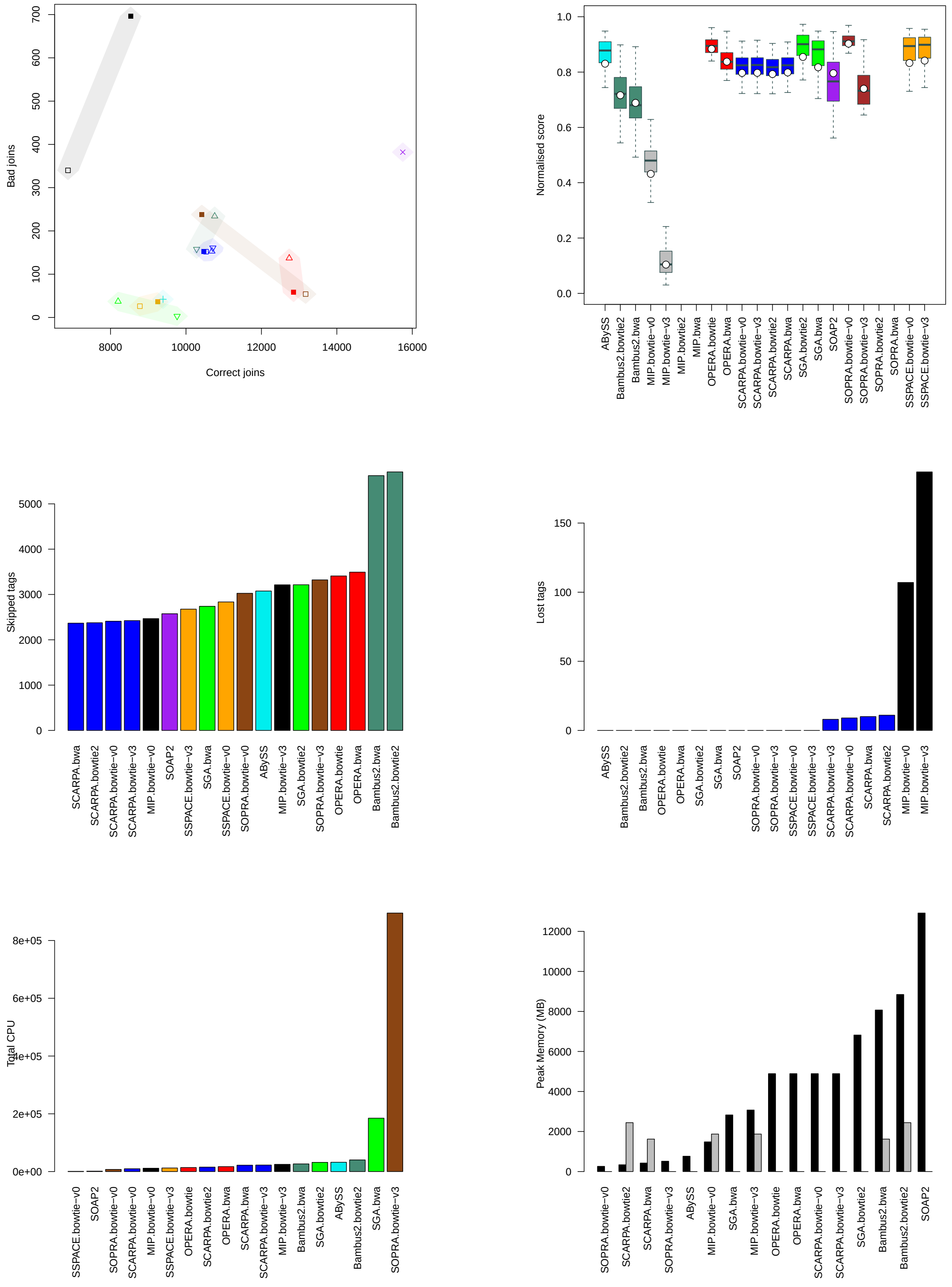

Figure 17: Results of scaffolding on the human chromosome 14 GAGE Velvet contigs using short and long fragment reads See the legend of Supplementary 6 for an explanation.
